# A tool for mapping microglial morphology, morphOMICs, reveals brain-region and sex-dependent phenotypes

**DOI:** 10.1038/s41593-022-01167-6

**Published:** 2022-09-30

**Authors:** Gloria Colombo, Ryan John A. Cubero, Lida Kanari, Alessandro Venturino, Rouven Schulz, Martina Scolamiero, Jens Agerberg, Hansruedi Mathys, Li-Huei Tsai, Wojciech Chachólski, Kathryn Hess, Sandra Siegert

**Affiliations:** 1 Institute of Science and Technology Austria (ISTA), Klosterneuburg, Austria; 2 Blue Brain Project, École Polytechnique Fédérale de Lausanne (EPFL), Geneva, Switzerland; 3 Department of Mathematics, KTH Royal Institute of Technology, Stockholm, Sweden; 4 Picower Institute for Learning and Memory, Massachusetts Institute of Technology, Cambridge, MA USA; 5 Department of Brain and Cognitive Sciences, Massachusetts Institute of Technology, Cambridge, MA USA; 6 Broad Institute of Harvard and MIT, Cambridge, MA USA; 7 Laboratory for Topology and Neuroscience, Brain Mind Institute, École Polytechnique Fédérale de Lausanne (EPFL), Lausanne, Switzerland; 8 Present Address: University of Pittsburgh Brain Institute and Department of Neurobiology, University of Pittsburgh School of Medicine, Pittsburgh, PA USA

**Keywords:** Microglia, Image processing, Alzheimer's disease, Glial development

## Abstract

Environmental cues influence the highly dynamic morphology of microglia. Strategies to characterize these changes usually involve user-selected morphometric features, which preclude the identification of a spectrum of context-dependent morphological phenotypes. Here we develop MorphOMICs, a topological data analysis approach, which enables semiautomatic mapping of microglial morphology into an atlas of cue-dependent phenotypes and overcomes feature-selection biases and biological variability. We extract spatially heterogeneous and sexually dimorphic morphological phenotypes for seven adult mouse brain regions. This sex-specific phenotype declines with maturation but increases over the disease trajectories in two neurodegeneration mouse models, with females showing a faster morphological shift in affected brain regions. Remarkably, microglia morphologies reflect an adaptation upon repeated exposure to ketamine anesthesia and do not recover to control morphologies. Finally, we demonstrate that both long primary processes and short terminal processes provide distinct insights to morphological phenotypes. MorphOMICs opens a new perspective to characterize microglial morphology.

## Abstract (web summary)

Colombo et al. build a morphological spectrum of over 40,000 microglia across development and disease with a topological data analysis approach that allows mapping of new conditions along these sex-region-specific and brain-region-specific atlases.

## Main

Morphological characterization of neuronal shapes has provided important insights into the diversity of cell types related to their genetic and functional features^1^. Numerous studies have tried to apply a similar morphological analysis on microglia^2–4^. Although they have revealed microglial heterogeneity^5–7^, no study has established a high-throughput, minimally biased and consistent way to capture context-specific and sex-dependent changes in microglial morphology during development and degeneration. Detecting subtle changes in the microglial morphology along the spectrum would offer an early readout of their immediate responses to local environmental cues^6^, as microglia are sensitive to changes in neuronal activity^8–10^. Moreover, the majority of these analyses rely on restricted microglia sample sizes underestimating their full morphological spectrum.

The microglia morphological phenotype is commonly determined with user-selected features from a three-dimensional (3D) reconstructed branching tree: these features can include total process length, branch number or number of terminal points. These scalar morphometric descriptors are then statistically compared across conditions. The drawback of this approach is the number and the type of selected features, which biases the biological readout: while too few selected features underrepresent a phenotypic difference, too many cause overfitting and introduce noise^11^. Moreover, in contrast to neuronal morphological trees that are static on the gross structure, microglia processes are highly dynamic^12,13^ as they constantly survey their local environment^12^. This introduces considerable intrinsic variability within the traced microglia population of a defined condition as well as the risk of selection bias to the extreme phenotypes. Establishing a reliable brain-region-specific morphological phenotype is critical for characterizing baseline morphology and tracking changes as deviations from the baseline.

To capture morphological phenotypes, complex morphological trees must be simplified with minimal information loss and retain as many features as possible. Applied topology provides new strategies for solving this problem, as it focuses on the shape properties of geometric objects without the need of morphometrics^14,15^. In particular, the topological morphology descriptor (TMD), which assigns a barcode to any 3D tree, has been successfully applied for classification of cortical neuron morphologies^14^. When we first applied the TMD to ~10,000 3D-traced microglia across the rostrocaudal axis of seven selected adult brain regions, these data indicated a regional phenotype, but the diversity of the individual microglia obscured any well-defined separation.

Here, we developed our MorphOMICs pipeline to overcome the major limitations of feature-selection-based analysis and biological data variability. MorphOMICs combines TMD with bootstrapping, dimensionality reduction and data visualization techniques, enabling minimally biased identification of the baseline phenotype. When we applied this strategy, we found that the morphologies of adult microglia vary between brain regions and are different between sexes. This microglial sexual dimorphism gradually diminished along postnatal development. In contrast, the sex-specific phenotype diverged during neurodegenerative disease progression, where females differ in their context-dependent response from males. When we aligned the trajectories of development and degeneration, we obtained for each brain region a morphological spectrum that we used as a reference atlas to map novel conditions. Remarkably, we resolved morphological changes after repeated exposure to the anesthetic cocktail ketamine–xylazine–acepromazine (KXA) and revealed that microglial morphology reverts away from the control during the recovery process. Our method unravels a spectrum of microglial phenotypes and overcomes the classical dichotomized view of either surveilling or reactive microglia. Thus, MorphOMICs lays out an avenue toward a multimodal definition of the microglia state.

## Results

### MorphOMICs uncovers adult microglial heterogeneity

**Fig. 1:**
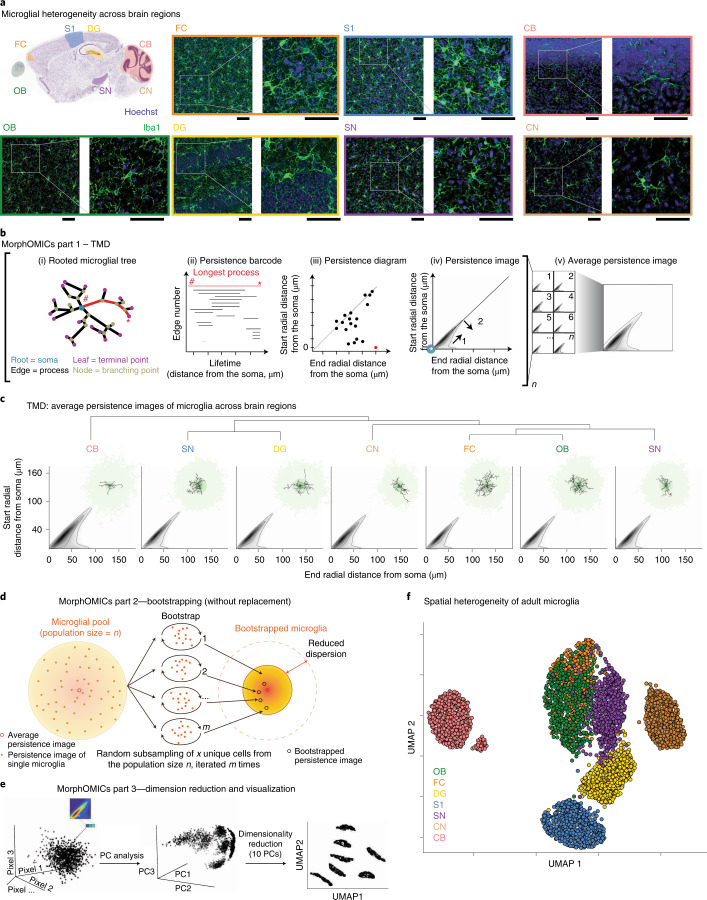
MorphOMICs dissects microglial morphology in adult healthy brains. **a**, Sagittal view of the mouse brain (image from Allen Institute) with annotated brain regions. Confocal images of immunostained microglia (Iba1, green) and cell nuclei (Hoechst, blue) from adult C57BL/6J mice with zoom-in view. Scale bars, 50 μm. **b**, Schematic of MorphOMICs pipeline covering the TMD with a mock microglia skeleton and plots. Red, longest process with start (#) and end (*). Each traced microglia wass converted into a rooted tree (i), followed by a persistence barcode (ii), a persistence diagram (iii) and a persistence image (iv) with grayscale process density in 2D space. Blue spot, soma location. Arrow 1 indicates the distance from the soma. Arrow 2 indicates the length of processes, which increases with distance from the diagonal. Each persistence image (*n*) is summarized to an average persistence image of a condition. **c**, Average persistence images of the seven analyzed brain regions organized by hierarchical clustering (Extended Data Fig. 1b). Top-right corner: representative traced microglia. The darker the green, the higher the frequency distribution of the processes. **d**, Schematic of MorphOMICs pipeline covering bootstrapping. Left: microglial population (*n*) contains individual persistence images. Center: average persistence image; *x* unique persistence images were drawn from each of *n* microglial pools to generate a bootstrapped persistence image. Right: repeating this process *m* times forms the bootstrapped pool. **e**, Schematic of MorphOMICs pipeline covering dimension reduction and data visualization with UMAP. Left: each persistence image is pixelated; each pixel represents a dimension. Middle: reducing dimensions with principal component (PC) analysis. Right: further dimensionality reduction based on the first ten PCs. **f**, UMAP plot of MorphOMICs-analyzed adult microglia, color-coded for each brain region. Each dot represents a bootstrapped persistence image. *n*_samples _= 500 per condition (‘Average and bootstrapped persistence images’). In **d**, see Supplementary Table 5 for the number of animals. Points situated close in the UMAP space indicate similar bootstrapped persistence image; however, the point’s actual position is irrelevant.

**Extended Data Fig. 1:**
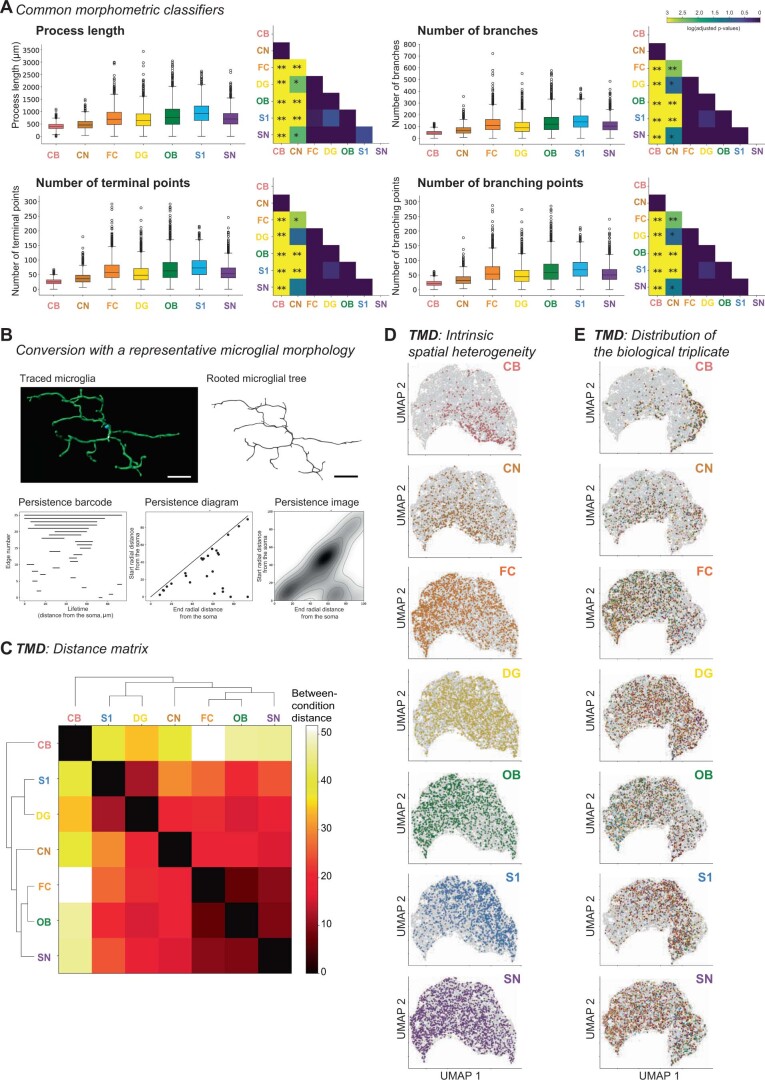
Classic morphometry analysis and intrinsic variability of microglial morphology. **a:** Box plots for the following morphometric features: process length, number of branches, and terminal and branching points for traced adult C57BL/6J microglia across different brain regions color-coded: CB (cerebellum, n = 299), CN (cochlear nucleus, n = 498), FC (frontal cortex, n = 926), DG (dentate gyrus, n = 902), OB (olfactory bulb, n = 796), S1 (somatosensory cortex, n = 719), SN (substantia nigra, n = 1050) from at least six animals. For number of animals per condition and region see also Supplementary Table 5. Box denotes first and last quartile with central line indicating the median. Whiskers: range of quantities. Open circle: outliers. Next, matrices with color-coded p-values for the pairwise comparison of each morphometric. Kruskal-Wallis test, Bonferroni correction using Dunn’s test, * p < 0.05, ** p < 0.01 (see Supplementary Table 1, 3). **b:** Example of a 3D-traced microglia and its conversion to a persistence image. Top: left, Imaris-traced skeleton of a microglia. Scale bar: 10 µm. Right, formatted rooted microglia tree, which is used for the persistence plots. Bottom: persistence barcode (left), persistence diagram (middle), and persistence image (right) exactly matching the rooted tree. **c:** Hierarchically-ordered heat map for pairwise TMD intrinsic distances between average persistence images from microglia across brain regions from Fig. 1a. **d**-**e:** UMAP plots of the entire microglial population size (grey) with color-highlighted brain regions (**d**) or animals (**e**). Each dot represents a single persistence image. (**e**) Triangle and circle for females and males, respectively. Each animal is color-coded.

To address how morphological phenotypes differ between microglia across brain regions, we immunostained the adult C57BL/6J mouse brain with the allograft inflammatory factor 1 (Aif1/Iba1)^16^ for both sexes with at least biological triplicates. Then, we traced 9,997 cells and generated a library of 3D microglial skeletons from seven brain regions chosen to span the rostrocaudal axis with a preference for regions that are known to be affected in Alzheimer disease^17–23^: the olfactory bulb (OB_mg_), frontal cortex (FC_mg_), dentate gyrus of the hippocampus (DG_mg_), primary somatosensory cortex (S1_mg_), substantia nigra (SN_mg_), cochlear nucleus (CN_mg_) and cerebellum (CB_mg_; Fig. 1a). When we utilized morphometrics that are commonly used in the field of microglial morphology^2,24,25^, we found non-significant differences across these brain regions with the exception of CB_mg_ and CN_mg_ (Extended Data Fig. 1a and Supplementary Table 1). We therefore applied the TMD^14,15^ for which each 3D skeleton was represented as a rooted tree with the microglial soma in the center, processes (edges), branching points (nodes) and process terminals (terminal points; Fig. 1b,i, i). The TMD converts the tree as a persistence barcode, where each bar represents the persistent process lifetime in terms of the radial distance from the soma (Fig. 1b)^11,14^. Every bar is then collapsed into a single point in the persistence diagram summarizing the process’s lifetime, which is then converted into a persistence image using Gaussian kernels^26^ (Fig. 1b). The branching complexity is spatially represented by process length proportional to the distance from the diagonal (Fig. 1b). An example of this conversion with a representative microglial morphology is shown in Extended Data Fig. 1b. To quantify the differences between microglial morphologies across brain regions, we computed the pairwise TMD distance between the average persistence images^14^. While average persistence images did not differ strongly (Fig. 1c), hierarchical clustering suggested groups with FC_mg_, OB_mg_, and SN_mg_, and S1_mg_ and DG_mg_ with CN_mg_ and CB_mg_ segregated (Extended Data Fig. 1c). When we looked at the individual persistence images, we found a wide variance between the individual microglia within a brain region, which made it challenging to distinguish regional phenotypes (Extended Data Fig. 1d). We note that this dispersion is not driven by an animal-based batch effect (Extended Data Fig. 1e).

**Extended Data Fig. 2:**
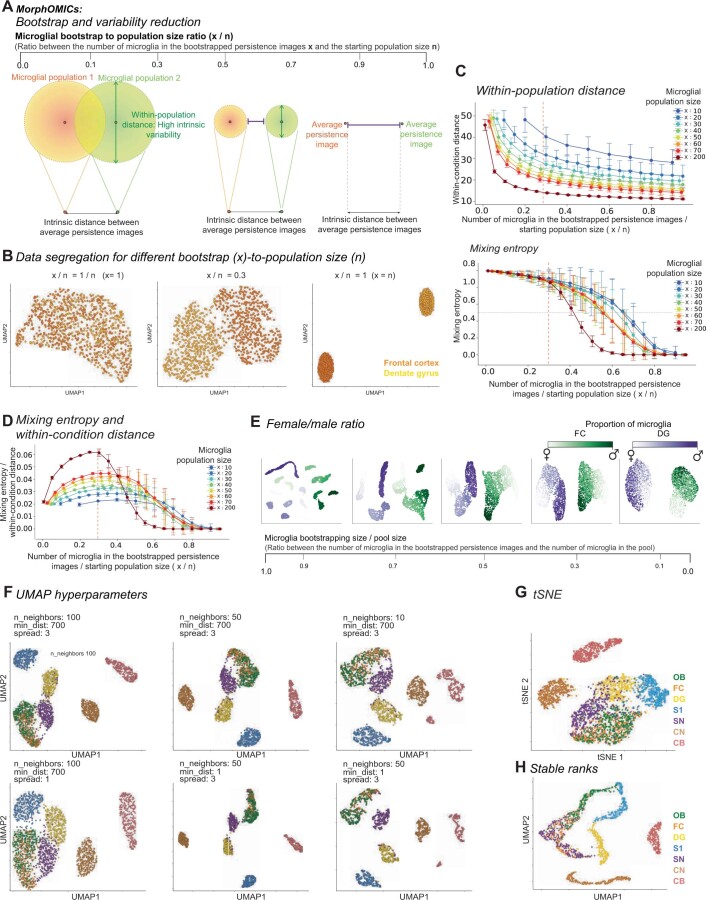
Details about the MorphOMICs paradigm. **a:** Schematic of the bootstrapping effects on the distance between tree structures from the same population (within-population distance, green arrows) and two distinct populations (distance between average persistence images, purple lines). Increase of bootstrap-to-population size ratio (x/n) reduces within-population distance and increases distance between average persistence images. **b:** UMAP plots of MorphOMICs-analyzed microglia for frontal cortex (orange) and dentate gyrus (yellow) for different bootstrap-to-population size ratios. Left: x = 1, allows no segregation. Middle: x/n = 0.3. Right: x/n = 1 causes accentuation. **c, d:** Line plot ± SD displays how within-population distance (**c**, top) and mixing entropy (**c**, bottom), and the ratio between mixing entropy and within-condition distance (**d**) varies by enhancing bootstrap-to-population size ratio (x/n). An empirical threshold of 0.3 was selected (red dashed line). Data are presented as mean values ± SD. **e:** UMAP plots of MorphOMICs-analyzed microglia for frontal cortex (green) and dentate gyrus (purple) for different bootstrap-to-population size ratios and varying male-to-female ratios within the population size. **f:** UMAP plots of MorphOMICs-analyzed microglial morphology across seven brain regions as shown for Fig. 1f with examples of tested hyperparameters for number of neighbors (n_neighbors), minimum distance (min_dist), and spread. Olfactory bulb (OB), frontal cortex (FC), dentate gyrus (DG), somatosensory cortex (S1), substantia nigra (SN), cochlear nucleus (CN), and cerebellum (CB). **g:** tSNE plot of MorphOMICs-analyzed microglia across seven brain regions as shown in Fig. 1f. **h:** UMAP plots of stable ranks representation of microglial morphology (see Methods: *Stable Ranks*) across seven brain regions. *n*_*samples*_ = 500 per condition (see also Methods: *Average and bootstrapped persistence images*). SD: standard deviation.

**Extended Data Fig. 3:**
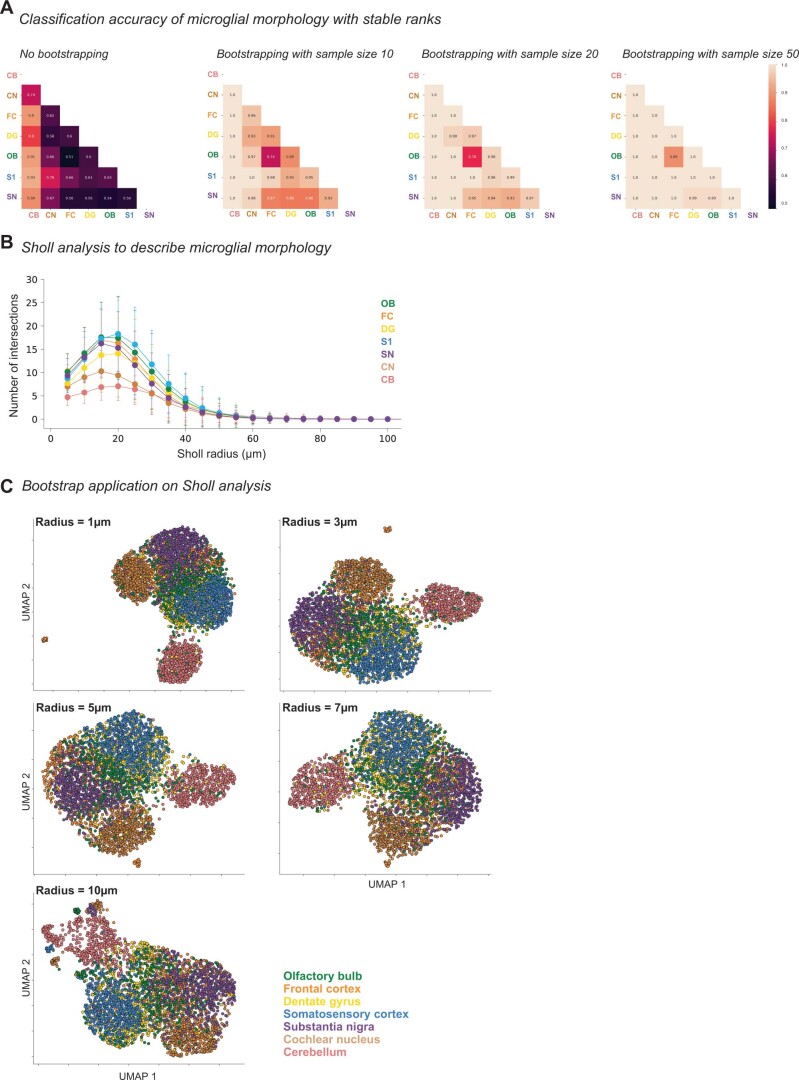
Classification accuracy of microglial morphology and bootstrap application on Sholl analysis. **a:** Heat-map of classification accuracy between pairs of brain regions using stable ranks for different bootstrap sizes. Numbers indicates the percentage of microglia correctly assigned in the classification task, averaged over 10 repeated cross-validations. 1, perfect assignment; 0.5 random assignment. **b:** Sholl curves for each brain region. Data are presented as mean number of processes (points) ± SD that intersect with a series of concentric Sholl spheres centered on the soma and spaced at 5 µm. For number of animals per condition and region see Supplementary Table 4, 5. **c:** Bootstrapped and UMAP visualized Sholl-analyzed microglia, color-coded for each brain region. Each dot represents a bootstrapped Sholl analysis. Each plot has a set radius step size (1, 3, 5, 7, 10 µm). Olfactory bulb (OB), frontal cortex (FC), dentate gyrus (DG), somatosensory cortex (S1), substantia nigra (SN), cochlear nucleus (CN), and cerebellum (CB). *n*_*samples*_ = 300 per condition (see also Methods: *Average and bootstrapped persistence images*). SD: standard deviation.

To overcome this intrinsic variability within a microglial population, we developed MorphOMICs, which combines TMD with subsampling of persistence images, dimensionality reduction and data visualization strategies. Bootstrapping randomly draws, without replacement, a user-defined number of unique persistence images (*x*) from a microglial population pool (*n*) and iteratively generates bootstrapped persistence images (Fig. 1d). To display these bootstrapped persistence images for each brain region, we applied the nonlinear dimensionality reduction technique uniform manifold approximation and projection (UMAP; Fig. 1e), which converts the high-dimensional persistence images into a reduced two-dimensional (2D) representation preserving their global structure. While local distances are presumably better preserved in UMAP compared to *t*-distributed stochastic neighbor embedding (*t*-SNE)^27^, the point’s actual position in the reduced space is irrelevant. After controlling for the bootstrapped-to-microglial population pool size ratio (Extended Data Fig. 2a–e and Supplementary Information), we applied MorphOMICs to our 3D-microglia library. The UMAP plot exhibited a spatial separation similar to that of the hierarchical clustering of the average persistence images (Fig. 1c), with CB_mg_ separated from the other brain regions and FC_mg_, OB_mg_ and SN_mg_ occupying a well-defined area in the UMAP space (Fig. 1f). However, MorphOMICs further revealed that OB_mg_ and FC_mg_ are intermingled, while DG_mg_ and S1_mg_ formed distinct clusters. Importantly, these cluster segregations were stable even if we changed UMAP’s hyperparameters (Extended Data Fig. 2f) or when we applied *t*-SNE visualization instead (Extended Data Fig. 2g). Finally, we also confirmed with stable ranks that the persistence barcodes maintained the region-specific phenotypes (Extended Data Fig. 2h). When we applied a support vector machine (SVM) algorithm to the stable ranks, the resulting classification accuracy confirmed the separation between brain regions in the UMAP space (Extended Data Fig. 3a). Notably, while the position of CN_mg_ varies with the choice of hyperparameters, its relative position to the other brain regions, especially DG_mg_ and SN_mg_, remains consistent. Thus, we suspect that more complex morphological relationships between brain regions can exist as exhibited by CN_mg_.

An alternative morphological simplification that is commonly performed in the microglia literature is Sholl analysis, which calculates the number of processes that intersect concentric spheres centered on the soma with a user-defined radius^28^. When we applied Sholl analysis, we could not recapitulate the spatial segregation captured by MorphOMICs (Extended Data Fig. 3b). Even if we applied bootstrapping to Sholl curves, we could only dissect the regional heterogeneity for CB_mg_ and CN_mg_ (Extended Data Fig. 3c). Moreover, these clusters merged with increasing Sholl radius step size. Overall, these data indicate that adult brain regions have well-defined microglia morphological phenotypes, which MorphOMICs reliably uncovers.

### Region-dependent, sexually dimorphic microglial phenotype

**Fig. 2:**
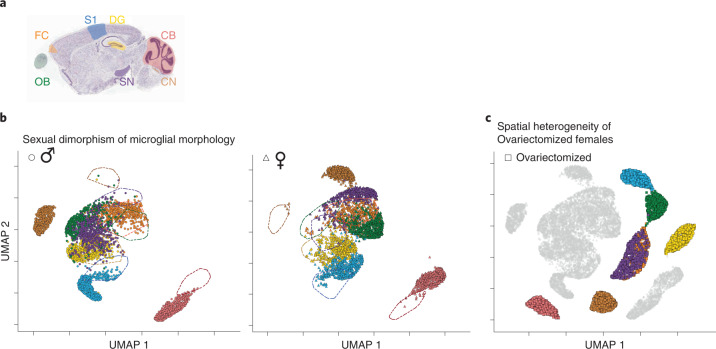
MorphOMICs identifies sexually dimorphic microglial morphology in healthy adults. **a**, Sagittal view of analyzed brain regions. **b**,**c**, MorphOMICs-analyzed microglia in male, female and ovariectomized adult mice. **b**, UMAP plot for each brain region color coded for males (left) or females (right) with dashed lines as reference. Each dot represents a bootstrapped persistence image. **c**, UMAP plot of ovariectomized females. Ovariectomized brain regions are highlighted. Gray indicates the non-ovariectomized counterpart as reference. *n*_samples_ = 500 per condition (‘Average and bootstrapped persistence images’).

**Extended Data Fig. 4:**
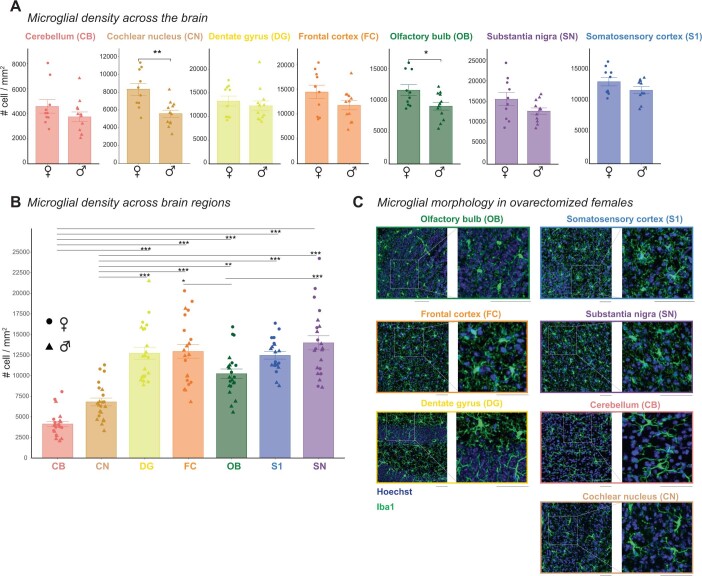
Adult microglial density and microglial morphologies after ovariectomy. **a**-**b:** Bar plot of microglial density distribution for each brain regions in C57BL/6J female and male adults. Data are presented as mean number of cells per mm^2^ ± SD. After determining normal distribution of the features with Shapiro-Wilk test, (**a**) sex averages for microglia from each region were compared with two-sided t-test. Each dot represents one animal. CN_mg_(n_♀_= 10, n_♂_= 12): t = 3.504, df = 15.1, p-value = 0.00312. OB_mg_(n_♀_= 10, n_♂_= 12): t = 2.401, df = 16.864, p-value = 0.0282. CB_mg_(n_♀_= 10, n_♂_= 12): t = 1.2564, df = 17.327, p-value = 0.2257. FC_mg_(n_♀_= 10, n_♂_= 12): t = 1.6236, df = 16.275, p-value = 0.1237. SN_mg_(n_♀_= 10, n_♂_= 12): t = 1.6261, df = 12.901, p-value = 0.1281. DG_mg_(n_♀_= 10, n_♂_= 12): t = 0.68892, df = 19.669, p-value = 0.4989. S1_mg_(n_♀_= 10, n_♂_= 12) t = 1.5618, df = 17.518, p-value = 0.1362.. SD: standard deviation. * p < 0.05, ** p < 0.01. (**b)** Analysis of variance (ANOVA) on densities yielded significant variation among conditions, F = 17.98, p < .001. F= 33.35, p < .001. Tukey post hoc test was computed for pairwise comparisons. (CN-CB)p.adj= 0.0523543; (DG-CB)p.adj= 0.0000000; (FC-CB)p.adj= 0.0000000; (OB-CB)p.adj= 0.0000000; (S1-CB)p.adj= 0.0000000; (SN-CB)p.adj= 0.0000000; (DG-CN)p.adj= 0.0000000; (FC-CN)p.adj= 0.0000000; (OB-CN)p.adj= 0.0036669; (S1-CN)p.adj= 0.0000002; (SN-CN)p.adj= 0.0000000; (FC-DG)p.adj= 0.9999824; (OB-DG)p.adj= 0.0898840; (S1-DG)p.adj= 0.9999557; (SN-DG)p.adj= 0.7953985; (OB-FC)p.adj= 0.0474778; (S1-FC)p.adj= 0.9985099; (SN-FC)p.adj= 0.9051447; (S1-OB)p.adj= 0.1998237; (SN-OB)p.adj= 0.0009832; (SN-S1)p.adj= 0.643197. Each dot represents one animal with symbols for female (circle) and male (triangle). Number of animals per region= 22. **c:** Confocal images of immunostained microglia (Iba1, green) and cell nuclei (Hoechst, blue) from ovariectomized C57BL/6J adult mice for each brain region with zoom-in. Scale bar: 50 μm. SD: standard deviation. Source data

Next, we were interested in the extent of microglial sexual dimorphism across brain regions, which is only partially understood^29,30^. We applied MorphOMICs to our library, and compared males and females within the UMAP space (Fig. 2a,b). As before, each brain region occupied a unique cluster in the plot, where CB_mg_ and CN_mg_ were most divergent. Strikingly, most brain regions separated female and male microglia, with CB_mg_, CN_mg_, OB_mg_, SN_mg_ and S1_mg_ forming close but spatially separated clusters. In contrast, ♂/♀DG_mg_ and FC_mg_ highly overlapped, suggesting rather minor morphological differences between the sexes. Interestingly, compared to Fig. 1c, the FC_mg_ and OB_mg_ cluster broke up: ♂FC_mg_ and ♂OB_mg_ formed spatially separated clusters, whereas ♀FC_mg_ and ♀OB_mg_ were intermingled. These morphological differences could depend on the microglia density. When we determined the number of microglia for each brain region and sex, we found that only CN_mg_ and OB_mg_ showed a significant sexual dimorphism (Extended Data Fig. 4a,b), which is also reflected in the strongest separation within the UMAP space (Fig. 2b). In contrast, microglia density does not explain the sexually dimorphic signature in CB_mg_ and SN_mg_ suggesting that density does not fully capture the dimorphic phenotype.

To determine whether these sex-specific phenotypic differences are hormone dependent, we expanded our library to include microglia from adult female mice that we ovariectomized at postnatal day (P) 20 (♀_ov_; Extended Data Fig. 4c) before they start the estrous cycle and enter puberty^31^. We found that the ♀_ov_FC_mg_ cluster no longer intermingled with ♀_ov_OB_mg_ in ovariectomized females but instead fused with ♀_ov_SN_mg_ (Fig. 2c). This is surprising, as in non-ovariectomized mice, ♀SN_mg_ was close to but distinct from the intermingled ♀FC_mg_ and ♀OB_mg_ cluster. When we compared non-ovariectomized to ovariectomized females, we found that in the UMAP space ovariectomized females formed distinct clusters, spatially separated from their non-ovariectomized counterparts and did not resemble any hints of masculinization (Fig. 2b,c). These results demonstrate the existence of a brain-region-specific, sexually dimorphic phenotype, and that interfering with estrogen production before puberty affects microglial heterogeneity in adulthood.

### Sexual dimorphism during development

**Fig. 3:**
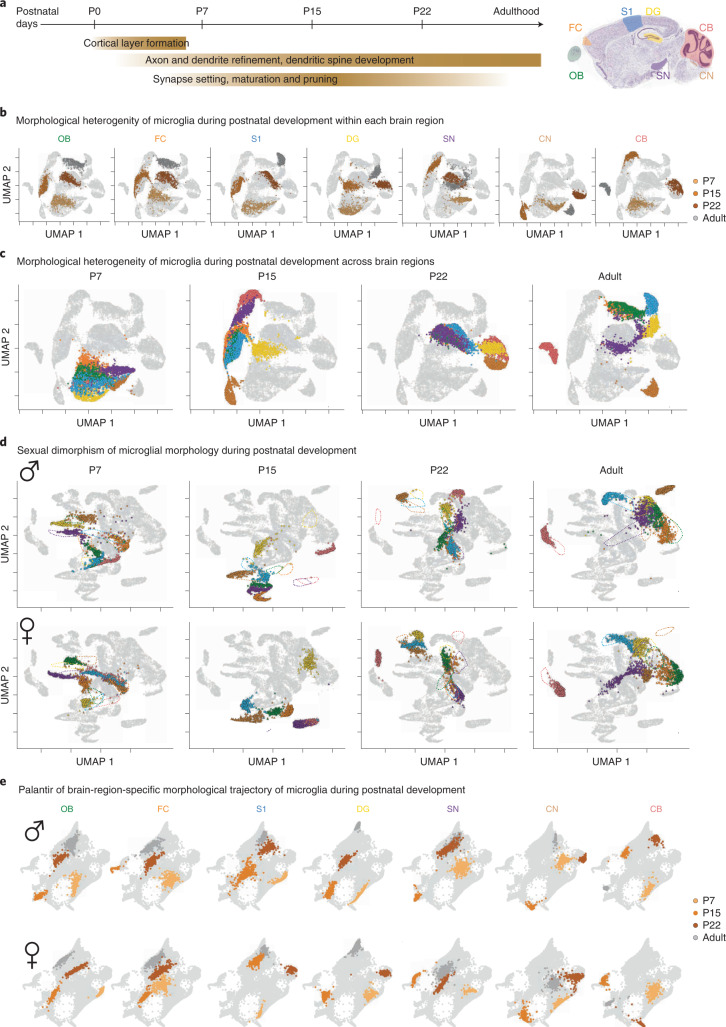
Microglial phenotypes during postnatal development. **a**, Timeline of postnatal brain development with highlighted events. Sagittal view of analyzed brain regions. **b**–**d**, UMAP plots of MorphOMICs-analyzed microglia across seven brain regions in *Cx3cr1*-GFP^+/−^ mice at P7, P15, P22 and adults (**b**), and color-coded brain regions for both sexes (**c**) and for each sex independently with dashed lines as the reference for each developmental time point (**d**). **e**, Palantir reconstruction of microglia morphological trajectory from **d** with P7, P15, P22 and adults highlighted for each brain region. Nearby points indicate similar persistence images. Each dot represents a bootstrapped persistence image. *n*_samples_ = 500 per condition (‘Average and bootstrapped persistence images’).

**Extended Data Fig. 5:**
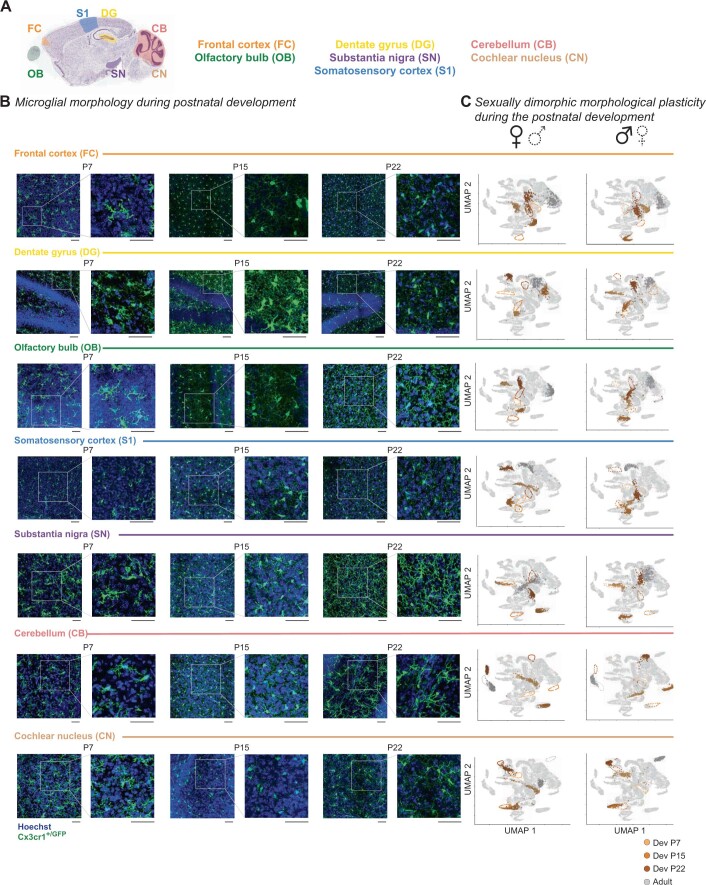
Microglial phenotypic spectrum during postnatal development. **a:** Sagittal view of analyzed brain regions: olfactory bulb (OB), frontal cortex (FC), dentate gyrus (DG), somatosensory cortex (S1), substantia nigra (SN), cochlear nucleus (CN) and cerebellum (CB). **b:** Confocal images of GFP^+^ microglia (green) and cell nuclei (Hoechst, blue) from Cx3cr1^+/GFP^ mice at P7, P15, and P22 for each brain region with zoom-in. Scale bar: 50 μm. **c:** UMAP plots of MorphOMICs-analyzed microglia from **b** for females (left) and males (right) at P7, P15, P22, and adults. Separate UMAP for each brain region and sex. Each dot represents a bootstrapped persistence image. *n*_*samples*_ = 500 per condition (see also Methods: *Average and bootstrapped persistence images*).

Microglia originate in the yolk sac and infiltrate the nervous system early during embryonic development^32^. After microglia occupy a brain region, their morphology gradually becomes more branched during postnatal neuronal circuit refinement (Fig. 3a)^33,34^. To determine whether microglial heterogeneity and the dimorphic phenotype already exist within the first postnatal weeks and before the onset of puberty, we sampled microglia from all seven brain regions at P7, P15 and P22 and included them in our library (Extended Data Fig. 5a,b). Then, we applied MorphOMICs and highlighted in the UMAP plots either each brain region (Fig. 3b) or the developmental time point (Fig. 3c). In all seven brain regions, no postnatal time points overlapped with the adult microglia (Fig. 3b), reflecting their morphological heterogeneity during development. When we analyzed each developmental time point individually, we found that at P7, all brain regions are distinct but occupy the same cluster, which shifted to a different cluster at P15 (Fig. 3c). Interestingly, CN_mg_ and DG_mg_ segregated and remained distinct from the other brain regions at P15 and P22, with CB_mg_ joining them at P22. Between P22 and adulthood, the clusters diverged to their adult microglial heterogeneity.

Next, we investigated whether sexual dimorphism affects the microglial phenotypic spectrum during development. To do this, we applied MorphOMICs to males and females separately (Fig. 3d and Extended Data Fig. 5c). Surprisingly, we found that the clusters shown in Fig. 3c split, leading to well-defined male and female clusters for each brain region at P7 (Fig. 3d). With brain maturation, ♀/♂ clusters in DG_mg_, FC_mg_ and S1_mg_ converged, while those in CB_mg_, CN_mg_, OB_mg_ and SN_mg_ remained distinct. To follow this sexual dimorphism along the developmental trajectory, we ordered the bootstrapped persistence images with the Palantir algorithm, which uses principles from graph theory and Markov processes to infer a pseudo-temporal trajectory (Fig. 3e). In the Palantir space, nearby points indicate similar persistence images, thereby assuming a gradual transition in their morphologies, and the continuous sequence of points define a trajectory. The developmental trajectories were similar between brain regions, with P7 and P22 clusters being the furthest from and the closest to the adult, respectively. In contrast, P15 shifted laterally from the P7–P22 trajectory and occupied the outermost position in nearly all the brain regions, indicating a unique microglial context-dependent response that coincides with neuronal circuit synapse refinement^35–37^.

### Link between morphology and response in the 5xFAD context

**Fig. 4:**
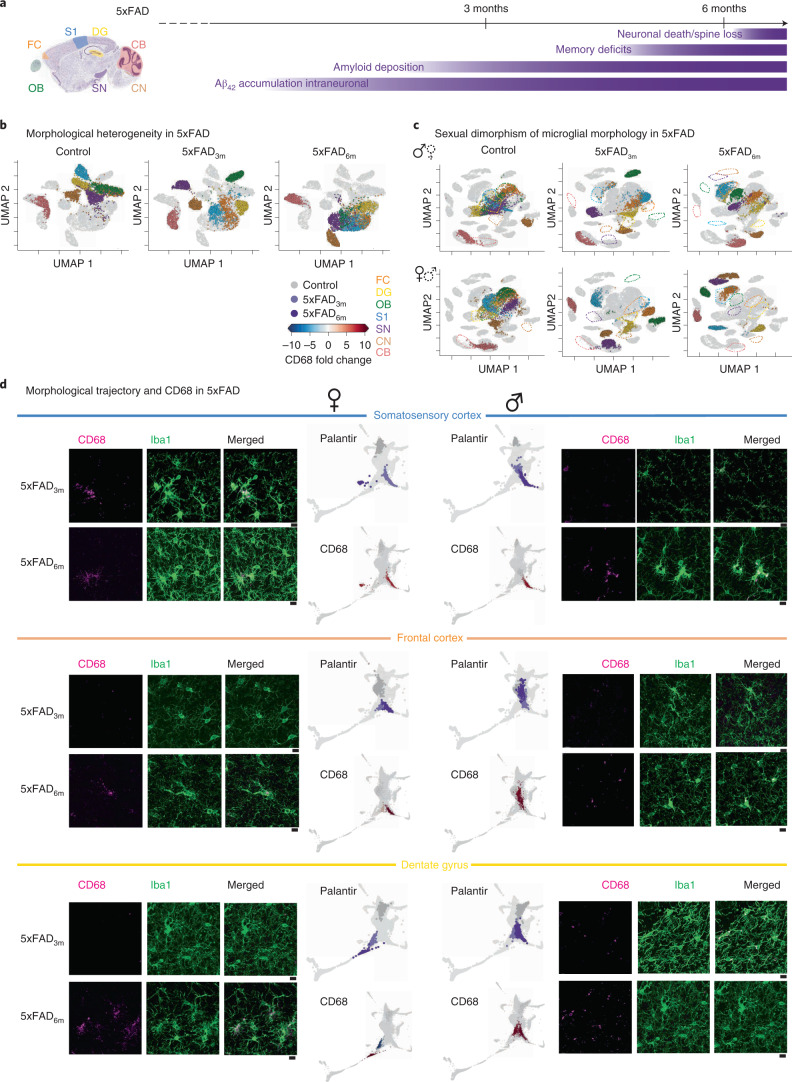
Microglia phenotypic spectrum in 5xFAD transgenic model of neurodegeneration is sexually dimorphic. **a**, Sagittal view of analyzed brain regions with color coding. Timeline of degeneration events in the 5xFAD transgenic mouse model. **b**,**c**, UMAP plots of MorphOMICs-analyzed microglia across seven brain regions (color coded) for control, 5xFAD_3m_ and 5xFAD_6m_ with both sexes (**b**) or for each sex separately (**c**). Each degeneration time point is highlighted in a separate UMAP. Each dot represents a bootstrapped persistence image. *n*_samples_ = 500 per condition (‘Average and bootstrapped persistence images’). **d**, Representative confocal images of immunostained microglia (Iba1; green) and lysosome (CD68; magenta), followed by Palantir reconstruction of microglial trajectory (top) with corresponding color-coded average CD68 fold change (bottom) across three animals from females and males for 5xFAD_3m_ and 5xFAD_6m_ in S1, FC and DG. Scale bar, 10 μm. Fold change < 0 in blue and >0 in red. Aβ42, amyloid beta.

**Extended Data Fig. 6:**
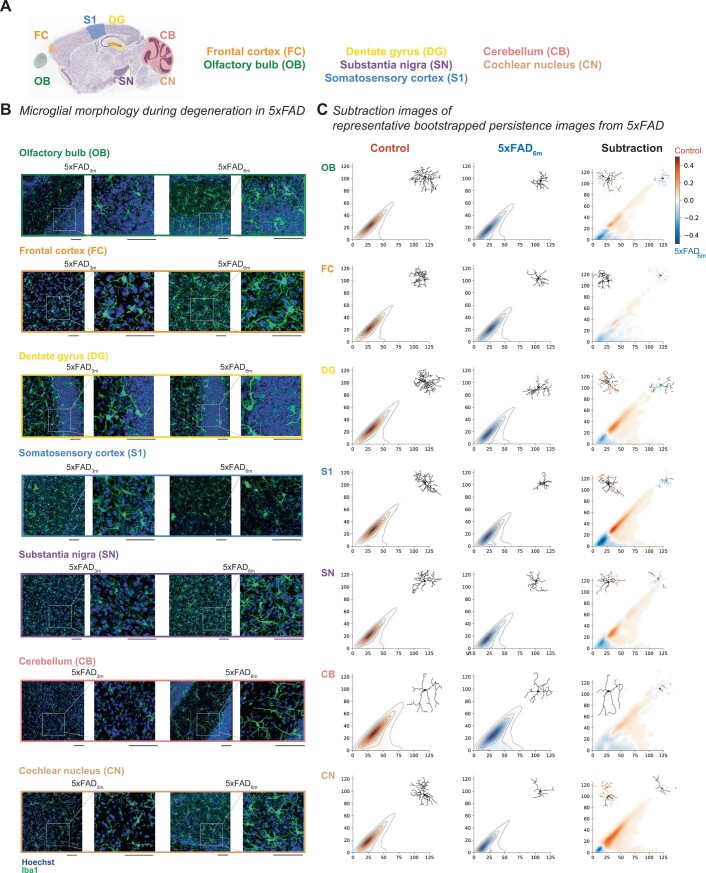
Microglia phenotypic spectrum in the 5xFAD model of familiar Alzheimer’s neurodegeneration. **a:** Sagittal view of analyzed brain regions: olfactory bulb (OB), frontal cortex (FC), dentate gyrus (DG), somatosensory cortex (S1), substantia nigra (SN), cochlear nucleus (CN) and cerebellum (CB). **b:** Confocal images showing immunostained microglia (Iba1, green) and cell nuclei (Hoechst, blue) from the analyzed brain regions in 5xFAD_3m_ and 5xFAD_6m_ (3 and 6 months, respectively) with zoom-in. Scale bar: 50 μm. **c:** Representative persistence images corresponding to each cluster centroid from Fig. 4b with color-coded process density. Top right corner, representative traced microglia. Subtraction image with highlighted overrepresented processes on the representative microglia.

Synaptic loss combined with amyloid plaque deposition are common signs of Alzheimer’s disease, with the neocortex and the hippocampus being the most affected brain regions^38^. Microglial morphology alters during the progression of Alzheimer’s disease^39^ but the disease phenotype of microglia in the directly and indirectly affected brain regions, as well as the impact on the sexual dimorphism, is not entirely understood^40,41^. To address this, we expanded our microglia with 3D-traced microglial morphologies in the 5xFAD mouse model (Fig. 4a), which recapitulates a familial form of Alzheimer’s disease^42^, for all seven brain regions. We focused on animals that were 3 and 6 months old (5xFAD_3m_ and 5xFAD_6m_, respectively; Extended Data Fig. 6a,b) because amyloid plaques occur first in the deep cortical layers at 3 months, followed by the hippocampus, coinciding with spine loss and memory deficits at around 6 months^42^. As anticipated, microglia in the 5xFAD_3m_ group exhibited a disease phenotype in which all brain regions were distinguishable from controls. The 5xFAD_6m_ group formed a ‘disease-associated cluster’ in the UMAP space, with the exception of CB_mg_ (Fig. 4b). S1_mg_, FC_mg_ and DG_mg_ already occupied this disease-associated cluster in 5xFAD_3m_. To obtain insights on which part of the microglia branching tree adapts during disease progression, we identified the representative bootstrap persistence image closest to the average for both control and 5xFAD_6m_ groups, and then subtracted them. Overall, the subtraction plots and the corresponding representative morphology indicated increased primary branches and loss of high-level branches in 5xFAD_6m_ compared to controls (Extended Data Fig. 6c). This effect was less pronounced in the FC_mg_ and CB_mg_ cluster, suggesting a brain-region-selective microglia reactivity that might adapt in a targeted way.

**Extended Data Fig. 7:**
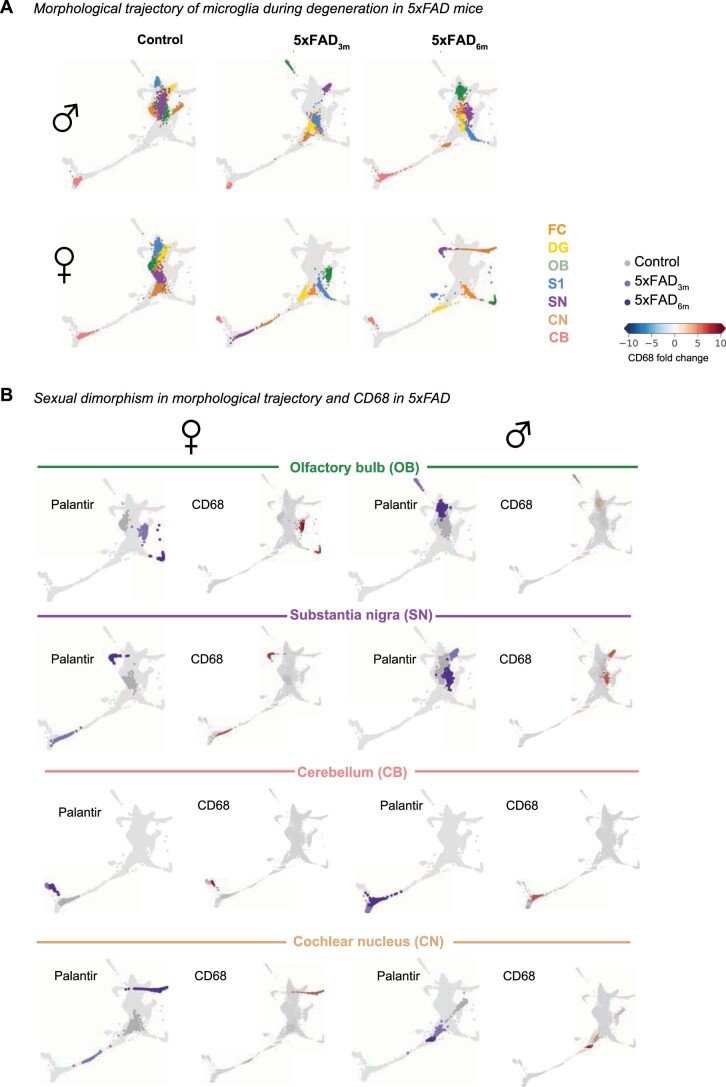
Sexually dimorphic microglia phenotype in 5xFAD. **a:** Palantir reconstruction of microglial morphological trajectory in males (top) and females (bottom). Each time-point highlighted in a separate Palantir plot. **b:** Palantir reconstruction of microglial trajectory with corresponding color-coded CD68 fold change next to it for females (left) and males (right) in control adult, 5xFAD_3m_, and 5xFAD_6m_ of OB, SN, CN, and CB. Fold change: blue < 0; red > 0. Olfactory bulb (OB), frontal cortex (FC), dentate gyrus (DG), somatosensory cortex (S1), substantia nigra (SN), cochlear nucleus (CN), and cerebellum (CB). *n*_*samples*_ = 500 per condition (see also Methods: *Average and bootstrapped persistence images*).

Next, we included the sex of the microglia in our MorphOMICs analysis. Microglia demonstrated higher morphological heterogeneity in 5xFAD_6m_ mice, with males partially overlapping and females spreading into clusters distinct from controls (Fig. 4c). When we applied Palantir to identify sex-dependent disease trajectories, we observed sexual dimorphism (Fig. 4d and Extended Data Fig. 7a), especially in one of the first affected brain regions, S1. ♀S1_mg_ seem to precede ♂S1_mg_:♀S1_mg_ clusters already overlapped in 5xFAD_3m_ mice with the trajectory that males reach only at 6 months (Fig. 4d). Such a difference was less obvious for FC_mg_ and DG_mg_, which is likely influenced by the limited number of selected time points during the pathology. Despite this, ♀DG_mg_ and ♀FC_mg_ display a phenotypic spectrum along the disease trajectory. To link microglial phenotype to their reactivity, we performed immunostaining for the endosomal/lysosomal marker CD68 (ref. ^43^). We then computed the fold change compared to the control CD68 volume within Iba1^+^ cells and overlaid the CD68 fold change on the Palantir trajectory (Fig. 4d). In ♀S1_mg_, CD68 increased already at 3 months, while this only occurred in ♂S1_mg_ at 6 months, confirming that the shift along the morphological spectrum happens earlier in females. For the other brain regions, this effect was less obvious. We also applied Palantir trajectories to the other brain regions, because plaque deposition has been reported in the olfactory bulb and brainstem^23^. We found a strong sexual dimorphism in microglial morphology in these brain regions, with less-obvious trajectory changes (Extended Data Fig. 7b). CB_mg_ was the only exception, remaining mainly unaffected in 5xFAD mice, which is consistent with previous literature^42^. Overall, the 5xFAD data indicate that the link between microglial disease phenotype and reactivity state depends on the brain region.

### Early shift of microglial morphology in female CK-p25 mice

**Fig. 5:**
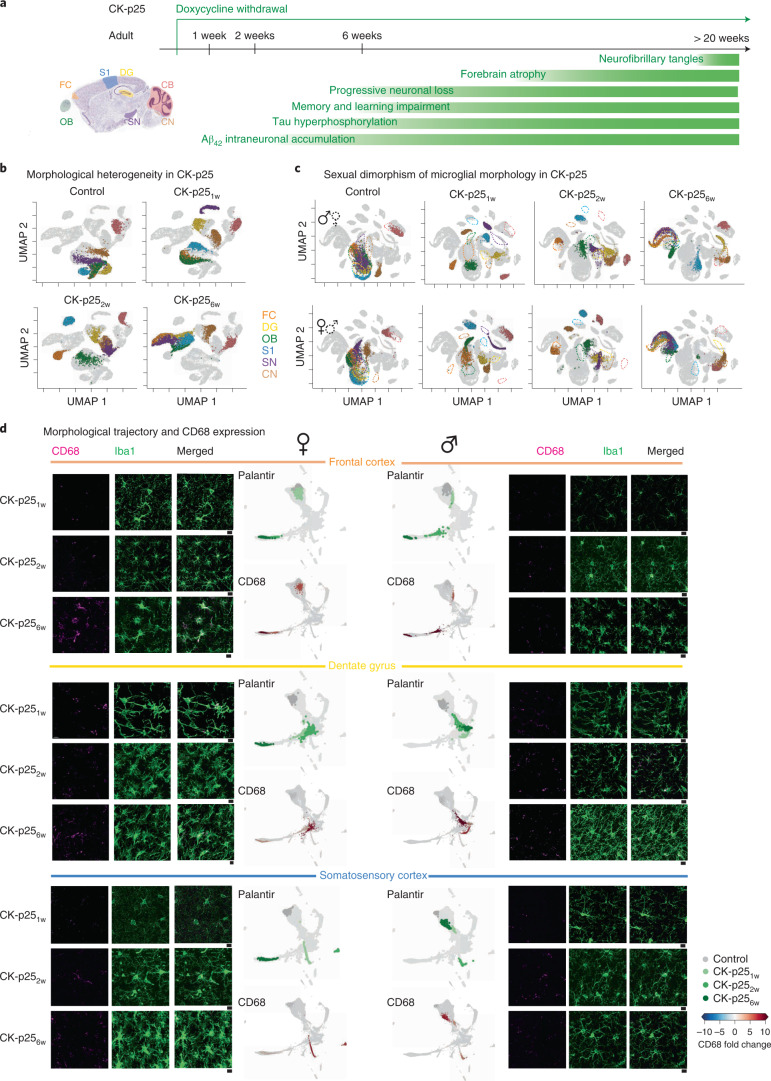
The microglia phenotype of females in CK-p25 model of neurodegeneration exhibit an earlier morphological shift than in males. **a**, Sagittal view of analyzed brain regions with color coding. Timeline of degeneration events upon doxycycline withdrawal in the CK-p25 transgenic mouse model. **b**,**c**, UMAP plots displaying microglial morphological heterogeneity in adult control mice and CK-p25 mice at 1, 2 and 6 weeks after doxycycline withdrawal across all the analyzed brain regions for both sexes (**b**) or for each sex separately (**c**). Each dot represents a bootstrapped persistence image, and each UMAP highlights a distinct degeneration time point. *n*_samples_ = 500 per condition (‘Average and bootstrapped persistence images’). **d**, Representative confocal images of immunostained microglia (Iba1; green) and lysosomes (CD68; magenta) in CK-p25 mice at 1, 2 and 6 weeks after doxycycline withdrawal in FC, DG and S1. Scale bar, 10 μm. Palantir reconstruction of microglial trajectory (top) with corresponding color-coded average CD68 fold change (bottom) across three animals. Females, left. Males, right. Fold change < 0 in blue and > 0 in red.

**Extended Data Fig. 8:**
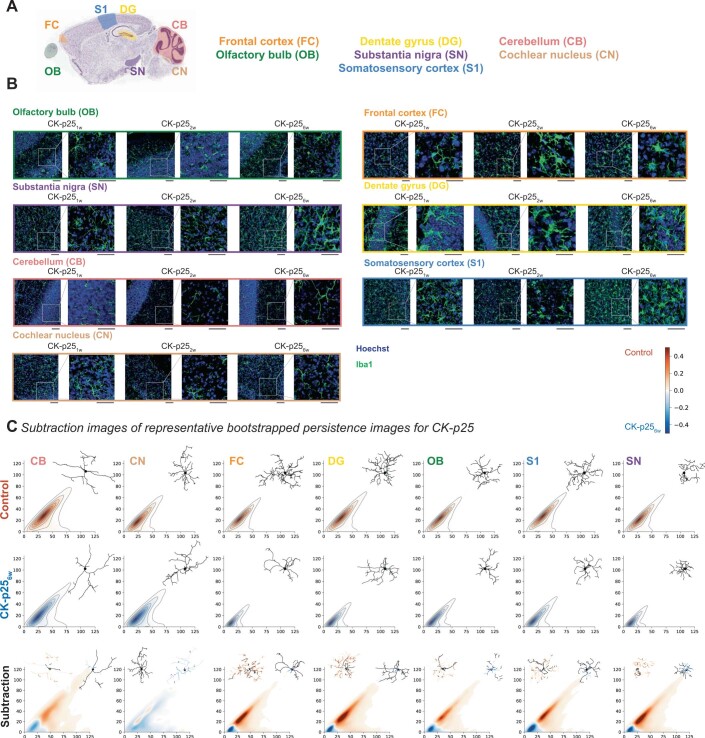
Microglia phenotypic spectrum in the CK-p25 model of sporadic neurodegeneration. **a:** Sagittal view of analyzed brain regions: olfactory bulb (OB), frontal cortex (FC), dentate gyrus (DG), somatosensory cortex (S1), substantia nigra (SN), cochlear nucleus (CN) and cerebellum (CB). **b:** Confocal images showing stained microglia (Iba1, green) and cell nuclei (Hoechst, blue) from analyzed brain regions and CK-p25_1w_, CK-p25_2w_, CK-p25_6w_ mice (1-, 2- and 6-weeks after doxycycline withdrawal) with zoom-in. Scale bar: 50 μm. **c:** Representative persistence images corresponding to each cluster centroid from Fig. 5b with color-coded process density. Top right corner, representative traced microglia. Subtraction image with highlighted overrepresented processes on the representative microglia.

An alternative model with faster onset and disease progression is the CK-p25 model for sporadic Alzheimer-like degeneration^44^. Upon doxycycline withdrawal, p25 expression is induced in CamKII^+^ forebrain neurons resulting in neurotoxic activity of the cyclin-dependent kinase Cdk6 (ref. ^45^). Within 2 weeks, CK-p25 mice develop progressive neuronal and synaptic loss, forebrain atrophy, aberrant amyloid-precursor protein processing, hyper-phosphorylation of tau and, at later stages, neurofibrillary tangle-like pathology^44^ (Fig. 5a). We reconstructed microglial morphologies from CK-p25 mice at 1, 2 and 6 weeks (CK-p25_1w_, CK-p25_2w_ and CK-p25_6w_, respectively; Extended Data Fig. 8a,b), included them in our library, and applied MorphOMICs. Similarly to 5xFAD, all seven brain regions started to segregate from the control at 1 week and occupied a disease-associated cluster in CK-p25_6w_, with CB_mg_ and CN_mg_ staying distinct (Fig. 5b). FC_mg_ reached this cluster already at 2 weeks, while OB_mg_, DG_mg_, S1_mg_ and SN_mg_ only at 6 weeks. When we obtained the subtraction plots between control and CK-p25_6w_, we found that microglia in CK-p25_6w_ have lost their high-level branches and their primary branches were overrepresented compared to control suggesting a reactive phenotype (Extended Data Fig. 8c). In contrast, CB_mg_ only showed a mild response likely due to an indirect effect as CaMKII is not expressed in the cerebellum. CN_mg_ morphology was mostly unaffected.

**Extended Data Fig. 9:**
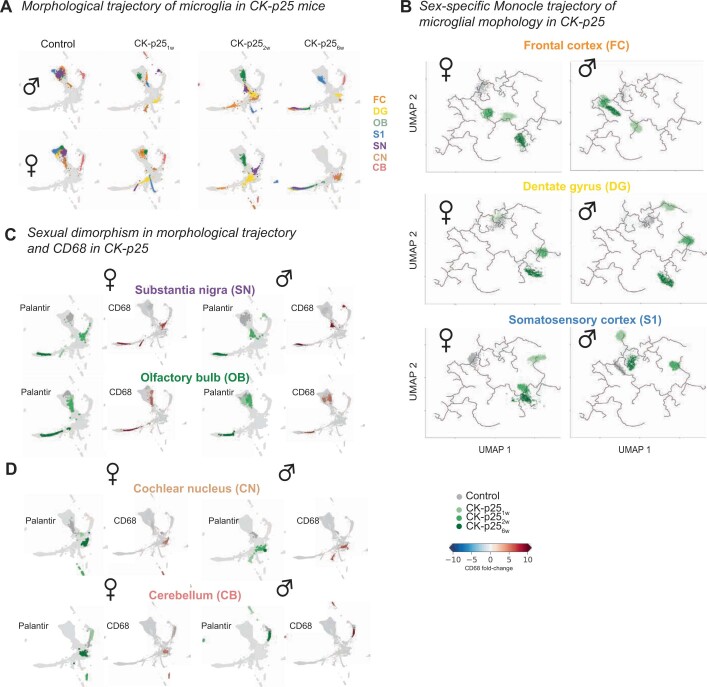
Sexually dimorphic microglial phenotype in CK-p25. **a:** Palantir reconstruction of microglial morphological trajectory in males (top) and females (bottom) in control and CK-p25 mice. Each disease time-point highlighted in a separate Palantir plot. **b:** Monocle reconstruction of microglia trajectory of females (left) and males (right) in control, adult and CK-p25 mice FC, DG, and S1. **c**-**d:** Palantir reconstruction of microglia trajectory and corresponding color-coded CD68 fold-change of females (left) and males (right) for SN, OB (**c**) and CN, CB (**d**). Fold change: blue < 0; red > 0. *n*_*samples*_ = 500 per condition (see also Methods: *Average and bootstrapped persistence images*).

Next, we applied MorphOMICs to the CK-p25 dataset separated by sex. We found that, in females, SN_mg_, FC_mg_, OB_mg_, DG_mg_ and S1_mg_ reached the disease cluster at 6 weeks, while in males, DG_mg_ and S1_mg_ stayed distinct (Fig. 5c). Similarly, Palantir displayed a trajectory arm, on which microglial morphology from later disease stages accumulated (Extended Data Fig. 9a). Neither CN_mg_ nor CB_mg_ reached this disease-associated arm as expected, due to the low expression of CaMKII in these brain regions^46^. Comparison of the sex-specific Palantir projections also showed that ♀FC_mg_ preceded ♂FC_mg_ in CK-p25_2w_ (Fig. 5d). We replicated the same dynamics with Monocle, an alternative algorithm which uses reversed graph embedding to infer a pseudo-time trajectory (Extended Data Fig. 9b)^47^.

When we overlaid the CD68 fold change compared to control adults over the Palantir FC_mg_ trajectory, we found that the CD68 fold change gradually increased (Fig. 5d), suggesting a CD68 dynamic that is different from morphological adaptations. Indeed, morphological changes did not correspond to CD68 in ♀DG _mg_ and ♀S1_mg_ at 6 weeks: the morphology reached the disease-associated arm but without increased CD68 fold change. Instead, ♂DG_mg_ and ♀DG_mg_ showed their highest CD68 fold change at 2 weeks and occupied a similar cluster in the Palantir space (Fig. 5d). Together, this suggests that the microglial response might be associated with the transient effect of p25 expression, which has been shown to enhance long-term potentiation and improve hippocampus-dependent memory, before inducing neurodegeneration, gliosis and severe cognitive decline at 6 weeks^48^. For those brain regions that were less affected, dimorphic microglial phenotype was less pronounced (Extended Data Fig. 9c). In both sexes, SN_mg_ and OB_mg_ in CK-p25_6w_ reached the disease-associated arm, whereas in CB_mg_ and CN_mg_, neither sex nor disease progression influenced morphology (Extended Data Fig. 9d). Overall, the CK-p25 model exhibited strong dimorphic phenotype spectrum in favor of females, which precede their male counterparts in a brain-region-specific manner.

#### Morphological information extraction with MorphOMICs

**Fig. 6:**
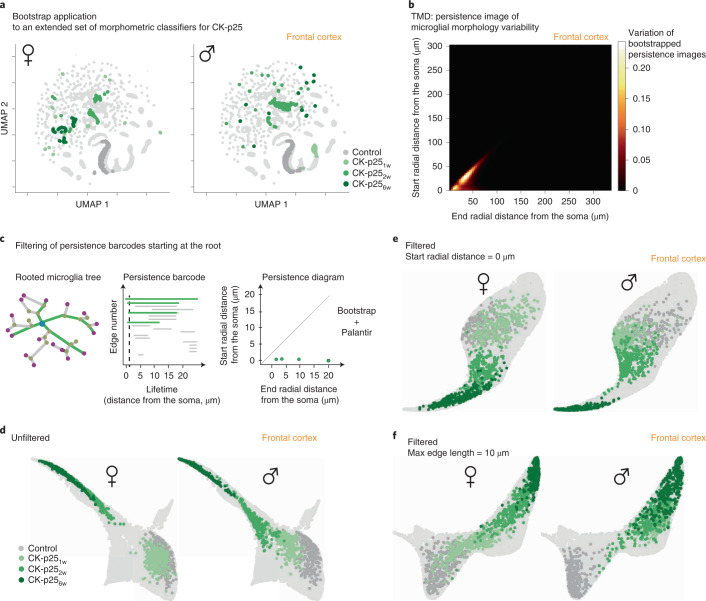
MorphOMICs applied to primary processes reiterates sexual dimorphism in CK-p25 mice. **a**, Bootstrapping and UMAP representations of an extended set of morphological classifiers (Supplementary Table 4) for control, CK-p25_1w_, CK-p25_2w_ and CK-p25_6w_ female and male mice across all brain regions (without cochlear nucleus and cerebellum for simplicity). The frontal cortex is highlighted. Each dot represents an averaged extended set of morphometric classifiers across 30 microglia that form the bootstrap population. **b**, Heat map of the bootstrapped persistence images pixel-wise standard variation across control and CK-p25 conditions of the frontal cortex. Black denotes no variation; white denotes high variation. **c**, Schematic for filtering persistence barcodes with MorphOMICs. Starting from microglial rooted tree, only bars are selected that are born at 0 µm independent of their length (representing likely primary branches, green), and are converted into a persistence diagram. **d**–**f**, Palantir trajectory of all brain regions (without cochlear nucleus and cerebellum for simplicity) from control and CK-p25 condition with highlighted FC microglia trajectory for females and males with unfiltered bars (**d**) or filtered bars (**e**, start radial distance from the soma, 0 µm; **f**, maximum bar length, 10 µm). *n*_samples_ = 300 per condition (‘Average and bootstrapped persistence images’).

**Extended Data Fig. 10:**
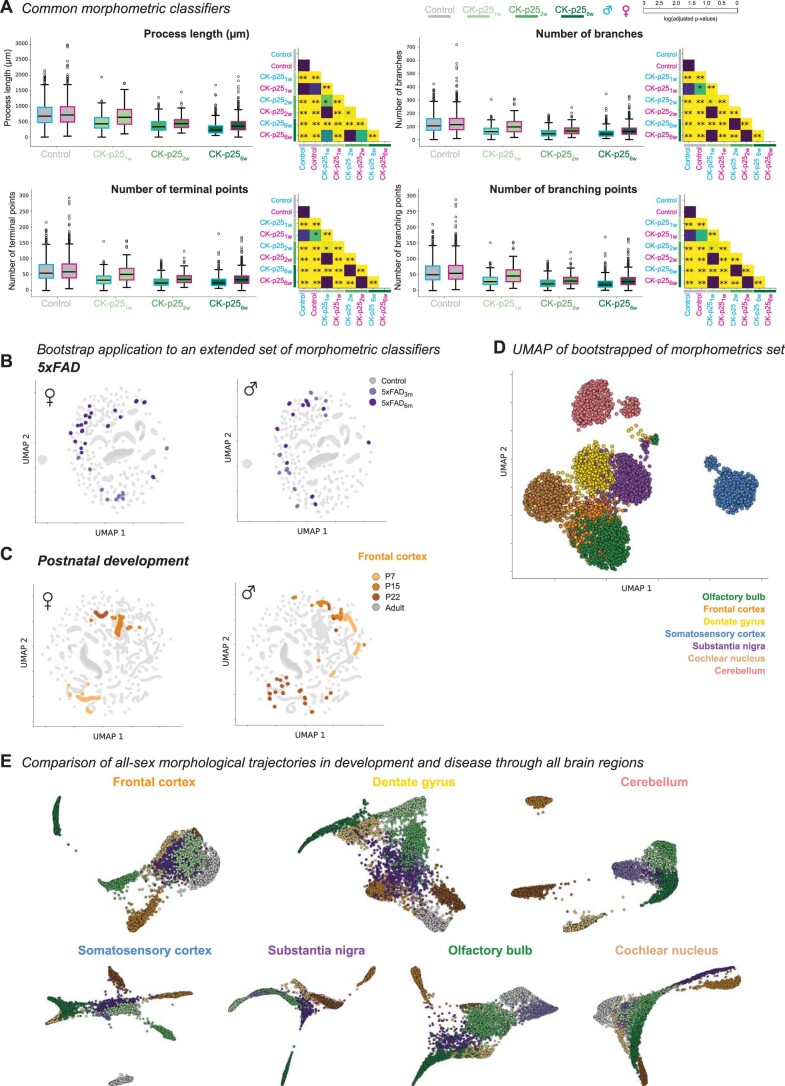
Classical morphometric do not recapitulate MorphOMICs observations. **a:** Box plots for the selected features process length, number of branches, terminal- and branching points of control (n_♀_= 926, n_♂_= 894), and CK-p25_1w_ (n_♀_= 219, n_♂_= 194), CK-p25_2w_ (n_♀_= 264, n_♂_= 492), CK-p25_6w_ (n_♀_= 858, n_♂_= 462) mice (1-, 2- and 6-weeks after doxycycline withdrawal, respectively) in the frontal cortex (FC). For number of animals per condition and region see also Supplementary Table 5. Box denotes first and last quartile with central line indicating the median. Whiskers: range of quantities. Open circle: outliers. Next: Matrices showing color-coded p-values for the pairwise comparison of each morphometric. Kruskal-Wallis test, Bonferroni correction using Dunn’s test, * p < 0.05, ** p < 0.01 (see Supplementary Table 1). **b**, **c:** Bootstrapped and UMAP representations of an extended set of morphological classifiers (see Supplementary Table 3) applied to females (left) and males (right) in 5xFAD (**b**) and developmental time points (**c**) with highlighted frontal cortex (calculation without cochlear nucleus and cerebellum for simplicity). Each dot represents an averaged extended set of morphometric classifiers across 30 microglia that form the bootstrap population. **d:** Morphometric UMAP of the bootstrapped comprehensive 27 morphometrics set showing regional heterogeneity of microglia in adult healthy mice. Brain regions are color-coded. **e:** Reference atlas for each brain region represented as Palantir reconstruction containing both sexes.*n*_*samples*_ = 300(**B**-**D**) and *n*_*samples*_ = 200 (**E**) per condition (see also Methods: *Average and bootstrapped persistence images*).

So far, we established both an adult sexual dimorphic microglia phenotype and a morphological spectrum during development and degeneration for seven brain regions. To further exemplify the superior performance of MorphOMICs over morphological feature selection, we applied common classifiers to the CK-p25 FC_mg_ dataset. Neither performing pairwise statistical comparisons of time points with common classifiers (Extended Data Fig. 10a and Supplementary Table 2) nor applying bootstrapping approaches to an extended set of non-interdependent morphometric quantities (Fig. 6a and Supplementary Table 3) replicated the sexually dimorphic control-to-disease spectrum from Fig. 5d. Similarly, we observed the same information loss for FC_mg_ in the 5xFAD model and during development (Extended Data Fig. 10b,c), although microglia from adult brain regions segregate (Extended Data Fig. 10d), suggesting that MorphOMICs preserves certain intrinsic properties of the reconstructed tree after dimensionality reduction.

To identify which properties are potentially relevant, we looked at the most variable pixels across CK-p25 FC_mg_ and the control bootstrapped persistence images (Fig. 6b). We found the highest variability along the diagonal and close to origin of the persistence diagram corresponding to short branches and branches close to the soma (Fig. 1b). We therefore decided to zoom in on the short and the long persistence bars, filtered them out separately, and repeated our MorphOMICs analysis (Fig. 6c–f). Using this method, we saw those bars corresponding to primary processes, sufficed to capture the sexually dimorphic phenotypes along the disease trajectory that we have previous seen (Fig. 6e). Interestingly, when we focused only on the short bars, reflecting short terminal processes, we found that males aggregated across all time points in a corner, whereas the females gradually adapted (Fig. 6f). These results suggest that persistence barcodes highlight different phenomena, and therefore both short and long bars are essential for the understanding of morphology.

### Atlas of context-dependent and cue-dependent microglial phenotypes

**Fig. 7:**
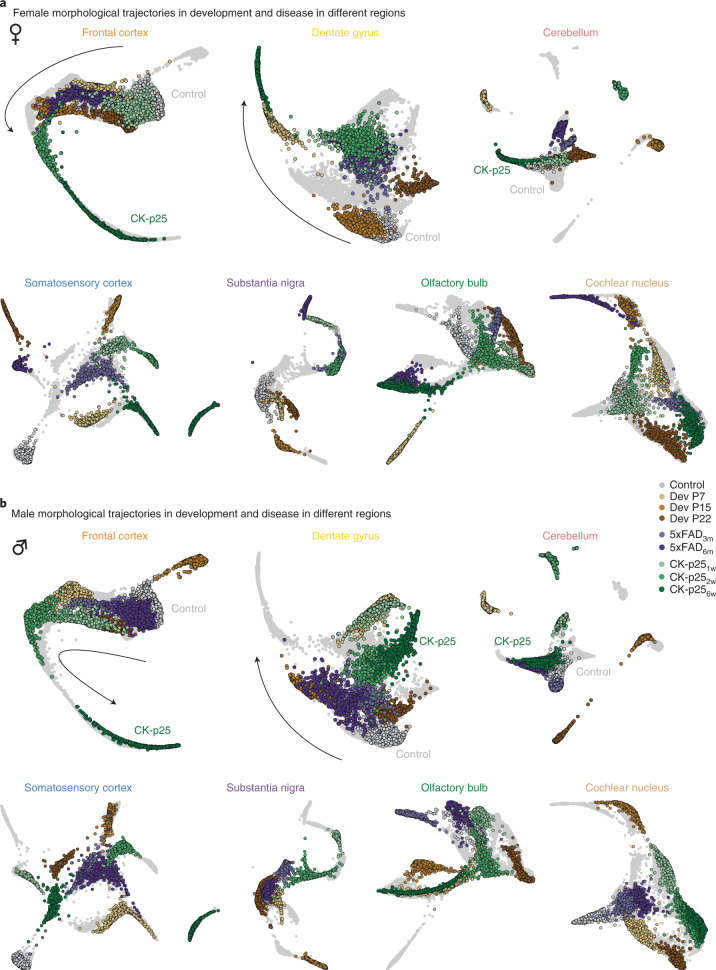
Development and disease phenotypes integrate into a reactivity spectrum. **a**,**b**, Palantir reconstructions calculated independently for each brain region for control, P7, P15, P22, CK-p25_1w_, CK-p25_2w_, CK-p25_6w_, 5xFAD_3m_ and 5xFAD_6m_ male and female mice. Microglial trajectory is highlighted for females (**a**) and males (**b**). Black arrow, control-to-disease spectrum. *n*_samples_ = 200 per condition (‘Average and bootstrapped persistence images’).

Until now, we have treated microglial morphology separately for development and disease. As both conditions induce a shift along the morphological spectrum, we were interested in how these conditions integrate along the pseudo-temporal trajectory to form a microglia reactivity spectrum. To achieve this, we performed MorphOMICs for each brain region and sex separately, including all developmental and disease time points and extracted the trajectory with Palantir (Fig. 7a,b). We first focused on the female reactivity spectra for FC_mg_ and DG_mg_ (Fig. 7a). In ♀FC_mg_, the P7, P15, P22, 5xFAD_3m_ and 5xFAD_6m_ groups aligned together reaching out toward the CK-p25_2w_ and CK-p25_6w_ groups, which extends away from all the other conditions, forming a disease-associated arm. Interestingly, ♀DG_mg_ mimicked a similar spectrum but with both 5xFAD and early CK-p25 forming a cluster distant from the P15 and the control, and the P7 group reaching out toward CK-p25_6w_. In both regions, microglia in 5xFAD_6m_ never reach the disease-associated arm suggesting a milder environmental condition compared to the late stages of CK-p25 neurodegeneration.

In males, disease phenotypes evolved more slowly than in females, with only ♂FC_mg_ reaching the disease-associated arm at CK-p25_6w_ (Fig. 7b). Like in females, we observed both 5xFAD groups close to the control together with P15, followed by intermingled P7, P22 and CK-p25_1w_. In ♂DG, microglial morphologies from P7 and CK-p25_1w_ clustered together, segregating from the rest of the conditions. Overall, ♂DG_mg_ displayed a similar phenotypic spectrum compared to ♂FC_mg_ for both 5xFAD groups, shifting toward the CK-p25_2w_–CK-p25_6w_ cluster. In the CB, both ♀ and ♂ CB_mg_, did not show any clear trajectory progression. Whereas the disease clusters mostly intermingled with the control, suggesting only a minor response to the disease environment, the developmental time points were distinct to the control (Fig. 7). Overall, our data show that microglia display a spectrum of phenotypes, with developmental time points occupying distinct parts of the trajectory in a brain-region-dependent manner.

### Morpho-functional relationship after repeated ketamine

**Fig. 8:**
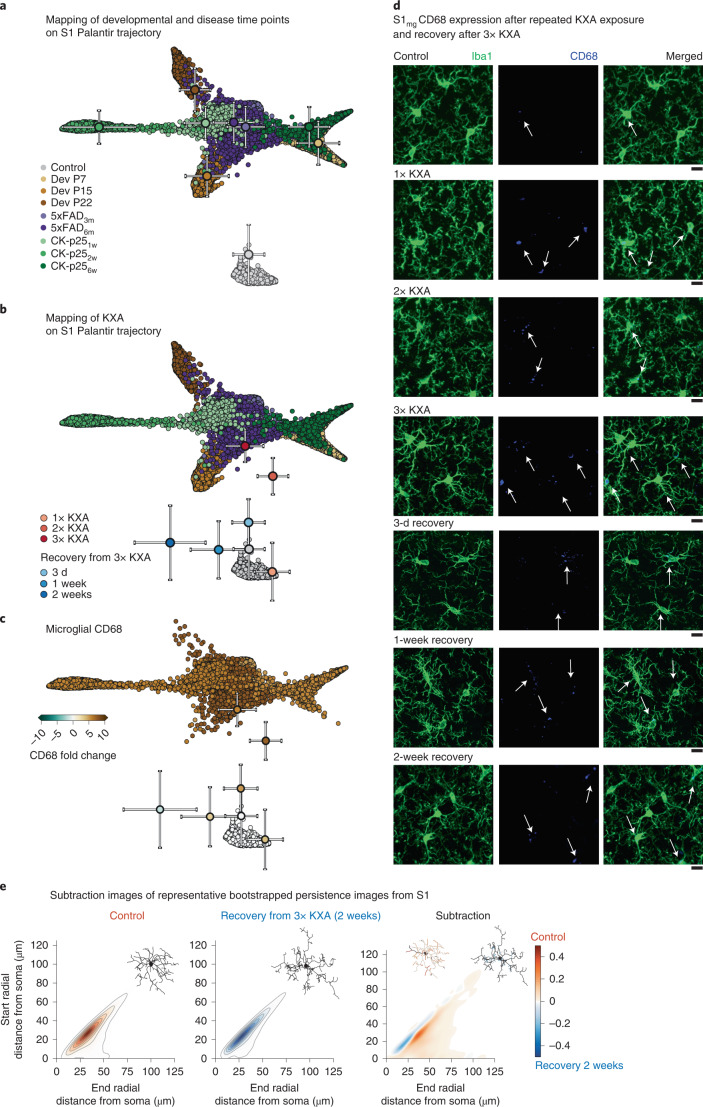
MorphOMICs applied to KXA-treated microglia. **a**, Mapping of S1 microglial morphology on Palantir trajectory. Centroids indicating the mean position of mapped points in a given condition with the corresponding standard deviations. **b**, Palantir reconstruction of microglia morphological trajectory in S1 from adult control, P7, P15, P22, 5xFAD_3m_, 5xFAD_6m_, CK-p25_1w_, CK-p25_2w_ and CK-p25_6w_ after 1×, 2× and 3× KXA and 3× KXA recovery after 3 d, 1 week and 2 weeks. Centroids indicate the mean position of mapped points in a given condition with the corresponding standard deviations. *n*_samples_ = 300 per condition (‘Average and bootstrapped persistence images’). **c**, Corresponding color-coded average CD68 fold change across three animals. Fold change, blue < 0; brown > 0. **d**, Representative confocal images of immunostained microglia (Iba1; green) and endosomal/lysosomal CD68 (blue) from control, 1×, 2× and 3× KXA and 3× KXA recovery after 3 d, 1 week and 2 weeks from the S1. Arrows indicate CD68 inside of microglia. Scale bar, 10 μm. **e**, Representative persistence images corresponding to control and 3× KXA_2w_ centroids from **b** with color-coded process density. Top-right corner: representative traced microglia. Subtraction image with highlighted overrepresented processes on the representative microglia.

We built a comprehensive library of 3D-traced microglia that, in combination with MorphOMICs, resolved a reactivity spectrum that can serve as a reference atlas for future addition of microglial morphologies (Extended Data Figs. 10e and 8a). To demonstrate the utility of such an atlas, we focused on S1 and used linear regression to estimate the location of the Palantir coordinates within a larger atlas with 2,000 bootstrapped persistence images per condition (Fig. 8a). As a first proof of concept, we generated new sets of bootstrapped images in all conditions and successfully mapped them to their corresponding clusters (Fig. 8b).

Next, we have recently shown a microglia-mediated extracellular matrix remodeling upon repeated exposure with the anesthetic ketamine^10^. Such treatment results in a dose-dependent microglia-mediated loss of the perineuronal net in the S1 and induces changes in plasticity^10^. However, we were not able to resolve morphological changes by eye (Fig. 8d). Thus, we used 3D-traced microglia in the S1 of adult C57BL/6J mice after 1×, 2× and 3× KXA treatment as well as after 3 d, 1 week and 2 weeks of recovery following the 3× KXA exposure, applied MorphOMICs and mapped their positions on the S1 reference atlas (Fig. 8b). Consistent with our previously reported dosage-dependent effect, 1× KXA did not lead to morphological adaptation, while with each additional dosage the microglial morphology gradually connected to the reactivity spectrum. In parallel, CD68 expression further upregulated with each additional dosage (Fig. 8c,d). This shows that MorphOMICs provides the fundaments for a morphology–function relationship, which could not be uncovered otherwise. Remarkably, MorphOMICs provides also a readout of microglial morphology adaptations following withdrawal after 3× KXA, which we have shown to reinstate the perineuronal net^10^. First, the morphology regresses toward the control phenotype after a 3-d recovery (Fig. 8b). Then, the microglial morphology separates on a trajectory, which diverges from the control and the KXA-treated ones suggesting a recovery-associated microglial phenotype. To gain insights on the morphological changes, we subtracted the control bootstrapped persistence image with the 2-week recovery (Fig. 8e). The recovery-associated microglia displayed more short processes closer to but not emanating from the soma, pointing toward a hyper-ramification. Overall, the combination of our reference atlas and MorphOMICs provides first insights into microglial morphology and their functional response.

## Discussion

In this study, we analyzed heterogeneity and sexual dimorphism of microglia morphology across seven brain regions from 41,872 cells through development, disease and under repeated ketamine exposure and recovery (Supplementary Tables 4 and 5). To establish a reference atlas of morphological phenotypes, we developed and applied the MorphOMICs pipeline, which extracts the information of the entire reconstructed microglial tree in a minimally biased way, combined with variability reduction and data visualization.

MorphOMICs takes advantage of applied topology and preserves the intrinsic properties of the reconstructed morphological tree avoiding feature-selection-derived biases. Contrary, classical morphometric classifiers include only user-selected features and there has been open debate about which classifier reliably describes the morphological phenotype of microglia. Recent studies have explored the options of alternative machine learning paradigms to identify phenotypic differences; however, they rely on a priori-labeled datasets and/or morphological feature selection^3,49,50^. MorphOMICs is independent of such assumptions. Furthermore, we demonstrate that commonly used morphometrics like process length, number of branches, terminal points or branching points failed to separate cells from different conditions (Extended Data Figs. 1a and 10a). Due to interdependency of these parameters, we expanded the list to 27 diverse classifiers (Supplementary Table 3)^51^ and applied bootstrap and dimensionality reduction, but even these parameters were insufficient to resolve differences in microglia morphology in CK-p25 (Fig. 6a), 5xFAD (Extended Data Fig. 10b) or postnatal development (Extended Data Fig. 10c). Similarly, Sholl curves could not fully recapitulate the spatial heterogeneity, and the radius step size influences the readout (Extended Data Fig. 3b,c). Interestingly, we found that both long primary processes and short terminal processes contain information that contribute to the microglial spectrum (Fig. 6c–f) emphasizing the importance of retaining as many features as possible. Classical morphometrics and Sholl curves are suboptimal for this.

Brain-region-specific single-cell transcriptome analyses have pointed toward microglial functional heterogeneity^52–54^, but morphological differences have so far been difficult to identify. MorphOMICs revealed that microglia in an adult brain exhibit regional heterogeneity (Fig. 1f) that exists already in early postnatal development (Fig. 3c) and diminishes during degeneration (Figs. 4b and 5b). Although microglia display a phenotypic spectrum (Fig. 7), they respond to diseases in a brain-region-dependent manner. Moreover, we confirmed that a sex-specific phenotype exists, which has long been debated^29^. This effect is rather mild during adulthood (Fig. 2b) but prominent during development (Fig. 3d) and degeneration (Figs. 4c and 5c). Both degeneration models showed a sexually dimorphic microglial response, which was pronounced in the immediately affected brain regions. Females showed an earlier shift along the morphological spectrum compared to males. This supports studies that have suggested a sex-dependent difference in Alzheimer’s disease progression^40,41,55^ and points to females having a higher risk of developing dementia^56,57^.

Estrogens have been shown to be involved in the masculinization of the brain^58,59^, and microglia are suspected of playing a role in this process^58^. Surprisingly, in the ovariectomized females, microglia are distinct from their non-ovariectomized counterparts and the brain regions intermingled differently (Fig. 2c). Whereas ♀FC_mg_ and ♀OB_mg_ occupied a similar cluster in control adults, ♀_ov_FC_mg_ were distinct from ♀_ov_OB_mg_ and highly intermingled with ♀_ov_SN_mg_ (Fig. 2c), suggesting that the impact of estrogens on microglial morphology is complex. Overall, MorphOMICs links the previously reported sexually dimorphic microglial transcriptome in the healthy brain^60–63^ and in degeneration models^64–68^ with a distinct morphological phenotype.

We showed that MorphOMICs detects microglial morphological changes at high resolution in several physiological conditions deviating from adult controls. For example, microglia shifted at P15 from the P7/P22 trajectory across all brain regions (Fig. 3e). This is the time of circuit refinement, where microglia have frequently been shown to participate in synaptic pruning^36,37^. Another synapse-associated pattern occurred in the DG_mg_ of CK-p25_2w_ (Fig. 5d). Unexpectedly, we found here the highest CD68 fold change and not within the CK-p25_6w_, where we have observed the most distinct morphological shift from the control. This discrepancy might be associated with previously observed transient p25 expression^48^. In general, we could not associate CD68 upregulation with a distinct microglia response phenotype.

MorphOMICs provides the fundaments to track subtle morphological changes that can be important indicators of local environmental changes and interference with the neuronal network. Whereas we have not found any obvious changes in the microglia morphology upon repeated ketamine anesthesia or the recovery (Fig. 8d), MorphOMICs overcomes the ambiguity and strongly predicts a microglia response based on our reference atlas (Fig. 8b) that can be investigated in a targeted manner. MorphOMICs provides an advanced strategy for systematically comparing microglial populations across different brain regions and conditions: this could be expanded infinitely. Future studies will focus on identifying informative regions of a persistence barcode, which provides a perspective for morphological analysis of lower-resolution images, such as in vivo microglial imaging for potential noninvasive diagnostic applications. Stable ranks would provide a mathematically robust approach to address this question, as we have shown that standard stable ranks of the TMD captured the microglial phenotypes (Extended Data Figs. 2h and 3a) as well as the persistence images of the microglial TMD (Fig. 1f). A critical point to consider is the number of cells that are needed for MorphOMICs. While we identified a suitable bootstrap size in Extended Data Fig. 2d, the condition-specific variability in microglial morphology needs to be systematically assessed to determine the minimum cell number before MorphOMICs can be reliably applied.

MorphOMICs overcomes the dichotomized view of microglial morphology to either ramified, relating to a surveilling function, or amoeboid, for participation in phagocytosis.

We anticipate that future studies will build on MorphOMICs and our atlas and will incorporate the epigenetic, transcriptional and/or proteomic landscapes along the microglial phenotypic spectrum. This will substantially advance our knowledge of the interplay between microglia and the nervous system.

## Methods

### Animals

C57BL/6J (no. 000664) and B6.129P-*Cx3cr1*^*tm1Litt*^/J (no. 005582, named here *Cx3cr1*^GFP/−^, only heterozygous were used) mice were purchased from The Jackson Laboratory. All animals were housed in the ISTA Preclinical Facility, under a 12-h light–dark cycle, with food and water provided ad libitum. Animals from both sexes were used. The number of animals used for each condition is detailed in Supplementary Table 5. All animal procedures were approved by the Bundesministerium für Wissenschaft, Forschung und Wirtschaft (bmwfw) Tierversuchsgesetz 2012, BGBI (I Nr. 114/2012, idF BGBI. I Nr. 31/2018 under the nos. 66.018/0005-WF/V/3b/2016, 66.018/0010-WF/V/3b/2017, 66.018/0025-WF/V/3b/2017, 66.018/0001_V/3b/2019 and 2020-0.272.234).

5xFAD and CK-p25 mice were obtained from the Tsai laboratory at Massachusetts Institute of Technology (MIT). All animal work was approved by the Committee for Animal Care of the Division of Comparative Medicine at MIT. 5xFAD mice (B6SJL-Tg(APPSwFlLon,PSEN1^*^M146L^*^L286V)6799Vas/Mmjax, stock no. 34840-JAX) were obtained from The Jackson Laboratory. CK-p25 mice^69^ were generated by breeding CaMKIIα promoter-tTA mice (CK controls) (B6;CBA-Tg(Camk2a-tTA)1Mmay/J, Jackson Laboratory, stock no. 003010) with tetO-CDK5R1/GFP mice (C57BL/6-Tg(tetO-CDK5R1/GFP)337Lht/J, Jackson Laboratory, stock no. 005706). CK-p25 mice were conceived and raised in the presence of doxycycline-containing food to repress p25 transgene expression. To induce p25 transgene expression, mice were fed a normal rodent diet. p25 transgene expression was induced in adult mice at the age of 3 months. For MorphOMICs, we compared CK-p25 brains upon drug withdrawal with our reference C57BL/6J adult microglia population. Notably, doxycycline withdrawal might affect the gut microbiome^70^, which can influence the microglial population in the brain and could cause some variability.

Mice were housed in groups of three to five on a standard 12-h light/12-h dark cycle, and all experiments were performed during the light cycle. Food and water were provided ad libitum.

### Brain samples and analyzed brain regions

We analyzed brains of both sexes from C57BL/6J adult mice (8–12 weeks) exposed to 1×, 2× or 3× KXA (100 mg per kg body weight ketamine, MSD Animal Health, A137A01; 10 mg per kg body weight xylazine, AniMedica, 7630517; 3 mg per kg body weight acepromazine, VANA, 18F211) and recovered 3 d, 1 week and 2 weeks after 3× KXA^71^; Cx3cr1^GFP/−^ mice at P7, P15 and P21; 5xFAD mice after 3 and 6 months; and CK-p25 mice 1, 2 and 6 weeks after doxycycline withdrawal. We focused on the following brain regions: the glomerular layer of the olfactory bulb (OB), cortical layer III–V of the frontal cortex (FC) and the primary somatosensory cortex (S1), the dentate gyrus of the hippocampus (DG), the substantia nigra (SN), the cochlear nucleus (CN) and the third lobe of the cerebellum (CB). The sagittal view of the brain sections analyzed (Figs. 1–5 and Extended Data Fig. 5a) was taken from the Allen Developing Mouse Brain Atlas-Sagittal and modified to show brain regions of interest^72^.

### Ovariectomy

Adolescent C57BL/6J females at P20 were anesthetized with 5% isoflurane in 0.5 l min^−1^ O_2_ during the anesthesia induction and 2% isoflurane in 0.5 l min^−1^ O_2_ during the maintenance phase. Using an electric razor, the fur was shaved to expose the skin over the lumbar spine and the region was sterilized with 70% (vol/vol) ethanol. A midline incision of approximately 1 cm was made on the skin in the lower back, below the chest. The subcutaneous tissue was gently dissected to expose the muscular fascia, and the ovarian fat pad was identified under the muscular layer. The peritoneal cavity was cut with a 0.5-cm incision. The Fallopian tube was exposed, and the ovary identified and cut at the level of the oviduct. The blood vessels were cauterized to prevent bleeding. The remaining part of the Fallopian tube was placed back in the peritoneal cavity, and the muscular fascia was sutured. The same protocol was repeated for the contralateral ovary. At the end, the skin was sutured. The animals received metamizole (Sanofi Aventis, no. Ay005, 200 mg per kg body weight during surgery) and meloxicam (Boehringer-Ingelheim, no. KPOEH3R, 5 mg per kg body weight subcutaneously after surgery every 24 h for 3 consecutive days), 2 mg per kg body weight subcutaneously after surgery. Animals were euthanized at P60.

### Transcardiac perfusion

For histological analysis, animals were quickly anesthetized with isoflurane (Zoetis, no. 6089373) and secured to the perfusion plate. The chest was open to expose the heart. The left ventricle was cannulated and the inferior vena cava cut. Animals were initially perfused with 30 ml of PBS with heparin (100 mg l^−1^; Sigma, no. H0878), followed by 30 ml of 4% (wt/vol) paraformaldehyde (Sigma, no. P6148) in PBS using a peristaltic pump (Behr, no. PLP 380, speed of 25 r.p.m.). Animals were decapitated, the brain explanted, fixed in 4% (wt/vol) paraformaldehyde for 30 min and post-fixed in 4% (wt/vol) PBS overnight (16 h). Then the tissues were washed in PBS and stored at 4 °C with 0.025% (wt/vol) sodium azide (VWR, no. 786-299). For cryoprotection, the tissue was transferred to 30% (wt/vol) sucrose (Sigma, no. 84097) in PBS and incubated overnight at 4 °C. To increase antibody permeability, the brain slices were frozen over dry ice and thawed at room temperature for three cycles.

### Vibratome sections

Cryoprotected samples were embedded into 3% (wt/vol) agarose/PBS to obtain coronal brain sections. The brain was sliced in 100-µm coronal sections on a vibratome (Leica VT 1200S).

### Immunofluorescence staining

The brain slices were incubated in blocking solution containing 1% (wt/vol) BSA (Sigma, A9418), 5% (vol/vol) Triton X-100 (Sigma, T8787), 0.5% (wt/vol) sodium azide (VWR, 786-299) and 10% (vol/vol) serum (either goat, Millipore, no. S26, or donkey, Millipore, no. S30) for 1 h at room temperature on a shaker. Afterwards, the samples were immunostained with primary antibodies diluted in antibody solution containing 1% (wt/vol) BSA, 5% (vol/vol) Triton X-100, 0.5% (vol/vol) sodium azide, 3% (vol/vol) goat or donkey serum, and incubated for 48 h on a shaker at room temperature. The following primary antibodies were used: rat α-CD68 (AbD Serotec, no. MCA1957, clone FA-11; 1807, 1:250 dilution), goat α-Iba1 (Abcam, ab5076, FR3288145-1; 1:250 dilution) and rabbit anti-Iba1 (GeneTex, no. GTX100042, no. 41556, 1 vol/vol 750). The slices were then washed three times with PBS and incubated for 2 h at room temperature on a shaker protected from light, with the secondary antibodies diluted in antibody solution. The secondary antibodies raised in goat or donkey were purchased from Thermo Fisher Scientific (Alexa Fluor 488 goat anti-rabbit IgG no. A11034, Alexa Fluor 647 goat anti-rat IgG 21247; 1:2,000 dilution). The slices were washed three times with PBS. The nuclei were labeled with Hoechst 33342 (Thermo Fisher Scientific, no. H3570; 1:5,000 dilution) diluted in PBS for 15 min. The slices were mounted on microscope glass slides (Assistant, no. 42406020) with coverslips (Menzel-Glaser no. 0) using an antifade solution (10% (vol/vol) Mowiol (Sigma no. 81381), 26% (vol/vol) glycerol (Sigma, no. G7757), 0.2 M Tris buffer (pH 8) and 2.5% (wt/vol) Dabco (Sigma, no. D27802)).

### Confocal microscopy

Images were acquired with a Zeiss LSM880 upright Airy scan or with a Zeiss LSM700 upright using a Plan-Apochromat ×40 oil-immersion objective 1.4 NA. Then, 2 × 2 *z*-stack tail images were acquired with a resolution of 1,024 × 1,024 pixels.

### Image processing

Confocal tile images were stitched using the software Imaris Stitcher 9.3.1.v. Then, the confocal images were loaded in Fiji 1.52e (http://imagej.net/Fiji). To remove the background, the rolling ball radius was set to 35 pixels, and images were filtered using a median 3D filter with *x*, *y* and *z* radii set at 3. Image stacks were exported as .tif files, converted to .ims files using the Imaris converter and imported into Imaris 8.4.2.v. (Bitplane Imaris).

### Quantification of CD68 volume within cells

Surface renderings were generated on microglia and CD68 *z*-stacks using the surface-rendering module of Imaris 9.2.v Surfaces were generated with the surface detail set to 0.2 µm. To determine the CD68 surface within microglia, the surface–surface coloc plugin was used. This analysis was performed on the entire image. The total ratio of CD68 volume within microglial volume (CD68-to-microglial volume) was calculated per image. To compute the CD68 fold change, the total CD68-to-microglial volume from each condition (sex/time point) was scaled to the CD68-to-microglial volume ratio from the respective adult control brain region. CD68 fold change > 1 means an increase in CD68 volume, while CD68 < 1 means a decrease in CD68 volume. CD68 fold change = 1 denotes no change in CD68 volume.

### Quantification of microglia density and statistical analysis

The spot-function plugin of Imaris 9.2.v was used to count the number of cells, that is, the soma of iba1-positive microglia within every confocal image. Microglial cell density was estimated as the total number of cells obtained in this way, divided by the size of the imaged sample in mm^2^.

### Reconstruction of 3D-traced microglia

After filtering and background subtraction, images were imported in Imaris 9.2.v (Bitplane Imaris). Microglial processes were traced in 3D with the filament-tracing plugin. Because the filament-tracing plugin provides a semiautomated reconstruction, this eliminates the need for a user-blind approach for selecting representative microglia. New starting points were detected when the largest diameter was set to 12 µm and with seeding points of 1 µm. Disconnected segments were removed with a filtering smoothness of 0.6 µm. After the tracing, we manually removed cells that were sitting at the border of the image and were only partially traced so that these cells would not be analyzed. The generated skeleton images were converted from .ims format (Imaris) to .swc format^73^ by first obtaining the 3D positions (*x*, *y* and *z*) and the diameter of each traced microglial process using the ImarisReader toolbox for MATLAB (https://github.com/PeterBeemiller/ImarisReader/) and then exporting for format standardization using the NL Morphology Converter (http://neuroland.org/). Artifacts from the 3D reconstructions automatically failed to be converted into .swc format.

### Analysis of morphometric features

Classic morphometric features were calculated from the .swc files using the functions Length (for total process length), N_branch (for number of branches), N_bifs (for number of branching points) and N_tips (for number of terminal points) from L-measure^74^ (http://cng.gmu.edu:8080/Lm/).

### Sholl analysis

Sholl curves were calculated from the .swc files using the sholl_crossings function of the NeuroM Python toolkit (https://github.com/BlueBrain/NeuroM). In brief, concentric Sholl spheres centered on the soma of a given traced microglia are constructed with a given step size radius. The number of microglial processes that intersect each Sholl sphere are determined. This step is performed for each traced microglia in the data. From this, Sholl curves of a microglial population are then calculated as the average number of intersections across the population.

### Topological morphology descriptor

A topological data analysis algorithm, the TMD, was used to extract topological phenotypes, called persistence barcodes, from 3D morphological structures (https://github.com/BlueBrain/TMD/;^14^). In brief, the 3D-reconstructed microglia is represented as a tree *T* rooted in its soma. The TMD summarizes this tree by calculating ‘persistence barcodes’, where each bar represents a persistent microglial process with respect to a filtering function, that is, the radial distance from the soma. Note that the persistence barcode that the TMD associates with *T* under this filtering function is invariant under rotations about the root and rigid translations of *T* in *R*^3^.

Each bar is described by two numbers: the radial distance, *d*_*i*_, at which a process originates; and the distance, *b*_*i*_, when it merges with a larger, more persistent process or with the soma. A bar can be equivalently represented as a point (*d*_*i*_, *b*_*i*_) in a ‘persistence diagram’. We could therefore convolve each point in the persistence diagram with a Gaussian kernel and discretize it to generate a matrix of pixel values, encoding the persistence diagram in a vector, called the ‘persistence image’.

### Average and bootstrapped persistence images

To construct the ‘average persistence image’ of a given condition, all the persistence barcodes of microglia from the same condition are combined before Gaussian convolution and discretization are performed. We also constructed average persistence images by performing first the Gaussian convolution and discretization of individual microglia persistence barcodes before taking the pixel-wise average. This produced qualitatively similar results.

The bootstrapping method subsamples the microglial population within a given condition, thereby introducing variations around the average persistence image. Starting from the population of all microglia from the same condition, called the ‘starting population’ of size *n* (Supplementary Table 4), the persistence barcodes of a predefined number of unique microglia, called the ‘bootstrap size’, are combined to calculate the ‘bootstrapped persistence image’. We iterated this process a predefined number of times, *n*_samples_, with replacement to obtain the ‘bootstrap sample’.

### Subtraction images and topological morphology descriptor distance

The subtraction image is the pixel-wise difference between two given persistence images. From the subtraction image, the TMD distance can be computed as the sum of the absolute pixel-wise difference. For stability of the TMD distance, we refer the reader to Kanari et al.^14^.

### Hierarchical clustering

Hierarchical clustering allowed us to find similarities between microglia across several conditions. Hierarchical clustering was done on the basis of the average persistence images. Clusters were then identified hierarchically using the average linkage criterion with the TMD distance metric and was implemented using cluster.hierarchy.linkage from SciPy v1.6.2 (https://www.scipy.org/). Dendrograms were generated using cluster.hierarchy.dendrogram to visualize the arrangement of the resulting cluster.

### Dimensionality reduction

#### Uniform manifold approximation and projection

A fast, nonlinear dimensionality reduction algorithm, UMAP^75^, was applied to visualize the high-dimensional pixel space of bootstrapped persistence images using a 2D representation while preserving local and global structures in the bootstrap samples (https://github.com/lmcinnes/umap/)^75^. Given a bootstrap sample containing multiple conditions, a TMD distance matrix containing pairwise distances between bootstrapped persistence images in the bootstrap sample is calculated. Principal components are then obtained using a singular value decomposition of the TMD distance matrix. The first seven principal components, where the elbow in the singular values is located, were used as input to UMAP with n_neighbors = 50, min_dist = 1.0 and spread = 3.0. Note that we tested for a wide range of parameter values that did not qualitatively change any of the aforementioned observations (Extended Data Fig. 2f).

#### *t*-SNE

An alternative dimensionality reduction algorithm is *t*-SNE (https://github.com/DmitryUlyanov/Multicore-TSNE/), which finds a dimensionality-reduced representation where similar points are pulled closer together while dissimilar points are pushed farther apart with high probability. The first seven principal components were taken as an input to run *t*-SNE with perplexity = 50.

#### Pseudo-temporal ordering

The concept of morphological phenotypes as encoded in the persistence images can be likened to transcriptional phenotypes in single-cell RNA-sequencing studies. Bootstrapped persistence images, which encapsulate morphological phenotypes of microglial populations from similar conditions, are comparable. Furthermore, it is reasonable to assume that morphological changes in bootstrapped microglial populations from control-to-disease conditions occur with incremental differences in the persistence images. This conceptual similarity allowed us to use the pseudo-temporal trajectory-inference algorithms that are well used in the single-cell RNA-sequencing community to study the morphological progression during microglial development and degeneration.

#### Palantir

Palantir^76^ uses principles from graph theory and Markov processes to calculate the pseudo-time and the probability of a cell reaching each of the terminal conditions in the sample (https://github.com/dpeerlab/Palantir/). First, the principal components of the bootstrapped persistence images were obtained using palantir.utils.run_pca with n_components = 100 and use_hvg = false. The diffusion maps were then calculated from the PCA projections using palantir.utils.run_diffusion_maps with n_components = 10 and knn = 20 which outputs the Palantir pseudo-times. Harmony^77^ was then used to construct an augmented affinity matrix from the Palantir pseudo-times to connect the Palantir pseudo-times and construct a trajectory using a force-directed graph (https://github.com/dpeerlab/Harmony/).

#### Monocle

To corroborate the Palantir trajectories, an alternative pseudo-temporal trajectory-inference algorithm called Monocle was used. Monocle^78^ uses reversed graph embedding, which learns a principal graph that approximates a lower-dimensional manifold to construct a pseudo-time trajectory (https://github.com/cole-trapnell-lab/monocle3/)^78^. Similar to Palantir implementation, the principal components of the bootstrapped persistence images were first obtained using preprocess_cds with num_dim = 100. A 2D UMAP representation was then obtained using reduce_dimension with umap.metric = ‘manhattan’, umap.min_dist = 1.0, and clusters were identified using cluster_cells with cluster_method = ‘leiden’. Finally, the pseudo-temporal trajectory was then obtained using learn_graph with use_partition = FALSE and close_loop = FALSE.

#### Stable ranks analysis

An alternative representation of the persistence barcodes is through stable ranks^79^. Stable ranks are functional summaries of persistence that depend on pseudometrics to compare persistence barcodes. Given a pseudometric *d*, the stable rank $\widehat {\mathrm{rank}_d}\left( X \right)\left( t \right)$ of a persistence barcode *X* is a function that assigns to *t* the number:

$$\widehat {\mathrm{rank}}_d\left( X \right)\left( t \right) = {\mathrm{min}}\left\{ {\mathrm{rank}\left( Y \right)|d\left( {Y,X} \right) \le t} \right\}.$$

whereby ‘rank(*Y*)’ denotes the number of bars of the persistence barcode *Y*. The stable rank $\widehat {\mathrm{rank}}_d\left( X \right)\left( t \right)$ associates to a persistence barcode a non-increasing and piece-wise constant function with values in [0, ∞). An important property is that this mapping is continuous with respect to the chosen pseudometric *d* and the *L*_*p*_ metric on the space ℳ of measurable functions.

A class of pseudometrics on persistence barcodes can be constructed from density functions^79^, which intuitively are used to vary the weight along the filtration scale parametrizing a barcode. With such pseudometrics, the stable rank is a bar count based on length of bars as scaled by the density. The standard stable rank is defined by a density function with constant value one. In this case, $\widehat {\mathrm{rank}}_d\left( X \right)\left( t \right)$ is the number of bars in *X* with length greater than or equal to *t*, that is, all filtration scales are weighted equally.

Stable ranks can be used in place of persistence images in the MorphOMICs pipeline. Similarly to MorphOMICs, the persistence barcode *X* of a given microglia is calculated using the TMD algorithm. To obtain ‘bootstrapped standard stable ranks’, we combined the persistence barcodes of a predefined number of microglia and computed their standard stable ranks. Dimensionality reduction was then implemented similar to the methods above (‘Dimensionality reduction’).

#### Classification accuracy using stable ranks

To support and quantify the impact of bootstrapping on the regional segregation visualized in the reduced UMAP space (Fig. 1f), we performed a classification task for microglia morphologies represented by their standard stable rank and labeled by brain region. We used an SVM with a specific kernel based on stable ranks^80,81^ for the classification. For persistence barcodes *X* and *Y*, the stable rank kernel with respect to a pseudometric *d* is given by

$$K_d\left( {X,Y} \right) = \mathop {\int}\limits_0^\infty {\widehat {rank}_d} \left( X \right)\left( t \right)\widehat {\mathrm{rank}}_d\left( Y \right)\left( t \right){\mathrm{d}}t.$$

where we used the pseudometric induced by the constant function with value one.

We performed pairwise classifications. For each pair of brain regions, we constructed a dataset consisting of 400 bootstrap samples, that is, 200 from each region and bootstrap sizes of 10, 20 or 50 (the results are reported separately for these three values). We randomly partitioned the dataset for cross-validation wherein 240 samples were used for SVM training (training set) and 160 samples for validation (test set). We reported the average accuracy over ten repeated cross-validations on the test set. The SVM was trained using the implementation in the Python library sklearn (https://scikit-learn.org/stable/) with default settings except for the usage of the stable rank kernel.

#### Bootstrapped morphometric features and bootstrapped Sholl curves

To understand whether classical morphology analysis pipelines are able to recapitulate the microglial dynamics recovered by MorphOMICs, a similar bootstrapping analysis was also done where we pooled a predefined number of microglia. Each morphometric quantity in the extended list enumerated in Supplementary Table 3 was then averaged to obtain a 27-dimensional vector, with each dimension corresponding to a morphometric feature, called the ‘bootstrapped morphometric features’. On the other hand, Sholl curves averaged across the pooled microglia to obtain the ‘bootstrapped Sholl curves’. Dimensionality reduction was then implemented similarly to the methods above (‘Dimensionality reduction’).

#### Mapping morphologies onto the reference atlas

We have generated a larger reference atlas with *n*_samples_ = 2,000 bootstrapped persistence images for each condition to construct the Palantir trajectories. Palantir coordinates (*x*, *y*) were rescaled to (0, 1). We took and filtered the bootstrapped persistence images keeping only the 500 most highly variable pixels across the images in all conditions. We used linear regression, one for each axis, to learn the mapping from the filtered bootstrapped persistence image to the rescaled Palantir coordinates. Given a novel condition, we generated the bootstrapped persistence images and filtered them with the 500 most highly variable pixels identified earlier. We used the trained regression model to infer the locations of each image in the reference atlas. Then, we calculated the mean position denoting the center of the inferred locations and indicated the spread using the standard deviation.

### Statistics and reproducibility

Each experiment was repeated independently with similar results. Supplementary Table 4 provides the numbers of 3D traces obtained per condition, sex and brain region. Supplementary Table 5 describes the number of animals per condition, their sex and brain region.

In Extended Data Figs. 1a and 10a, statistical analysis was performed using scipy.stats (v1.6.2) and scikit-posthocs (v0.6.7). These morphometric features were first tested for normality using the Kolmogorov–Smirnov test (scipy.stats.kstest). After determining the non-normal distribution of the features, we performed non-parametric pairwise tests for independence between measurements from two brain regions using the Kruskal–Wallis test (scipy.stats.kruskal). We used Bonferroni-corrected *P* values, calculated using Dunn’s test via scikit_posthocs.posthoc_dunn (Supplementary Table 1).

In Extended Data Fig. 4a,b, statistical analysis was performed using R (v3.4.4). Normality of the microglial average densities was tested using Shapiro–Wilk’s test (shapiro.test()). Differences in densities were compared with two-sided *t*-test. When comparing between brain regions, Tukey’s post hoc test (TukeyHSD()) was used for multiple comparisons (Supplementary Table 2).

### Reporting summary

Further information on research design is available in the Nature Research Reporting Summary linked to this article.

## Online content

Any methods, additional references, Nature Research reporting summaries, source data, extended data, supplementary information, acknowledgements, peer review information; details of author contributions and competing interests; and statements of data and code availability are available at 10.1038/s41593-022-01167-6.

## Supplementary information

Supplementary Information Supplementary Discussion and Tables 1–5.

Reporting Summary

## Data Availability

The .swc files generated during the current study are available in the NeuroMorpho.org repository in the Siegert archive at https://neuromorpho.org/KeywordResult.jsp?keywords=%22siegert%22. Source data are provided with this paper.

## Source data

Source Data Extended Data Fig. 4 Source data for generating the bar charts in Fig. 4a,b.

## Code availability

The codes and the reference atlas are available to download on GitHub (https://git.ist.ac.at/rcubero/morphomics/), with detailed instructions on implementation.
