# Supplementary material for: A tool for mapping microglial morphology, morphOMICs, reveals brain-region and sex-dependent phenotypes
Source: Nat Neurosci. 2022 Sep 30;25(10):1379–93. doi: 10.1038/s41593-022-01167-6 (PMC9534764; doi:10.1038/s41593-022-01167-6)
Supplement: Supplementary file 1 — Supplementary Discussion and Tables 1–5. [file 41593_2022_1167_MOESM1_ESM.pdf]

nature neuroscience

Resource

https://doi.org/10.1038/s41593-022-01167-6

# A tool for mapping microglial morphology, morphOMICs, reveals brain-region and sex-dependent phenotypes

In the format provided by the authors and unedited

## Supplementary text

### *Computational assessment of bootstrapping methods in MorphOMICs*

#### *1. Single-condition case*

Microglia are highly dynamic [1] – a feature which is inherent to their function. Under homeostatic conditions, microglia survey their local environment for insults and anomalies. This intrinsic variability challenges the topological analysis of microglial morphology as we observed in the corresponding persistence images of single microglia (**Fig. 1B**). This variability can mask heterogeneity between microglial populations from different biological conditions *e.g.*, brain region, sex, and development and disease time point (**Extended Data Fig. 1C**).

To overcome this intrinsic variability within microglial populations, we use bootstrapping methods. Bootstrapping is a statistical method that combines random resampling and permutation. It is commonly used to calculate standard errors, to construct confidence intervals, and to perform hypothesis testing for numerous types of sample statistics. In our case, we used bootstrapping to randomly pool together a pre-determined number of microglia within a condition, called the bootstrap size, to reduce the dispersion. This allowed us to construct a bootstrapped persistence image of this microglial sub-population. By averaging out the highly variable portions of the persistence images, we retain the topological signatures that may separate different conditions. Moreover, bootstrapping makes it possible to create as many bootstrapped persistence images as desired.

The pixels of the persistence image span a high-dimensional space. The bootstrapped persistence images form a point cloud in this high-dimensional space with the average persistence image in the center (**Extended Data Fig. 2A**). Thus, the spread of this cloud allows us to assess the variability within a microglial population. Intuitively, the bootstrap size affects this point cloud size. To construct the bootstrapped persistence images with just a single microglia will give us the largest cloud as it reflects the full size of the population's dispersion. On the other hand, when the bootstrapped persistence image is constructed using all of the microglia, the cloud collapses to a single point as there is no difference between the bootstrapped persistence images and the average persistence image.

To systematically understand the effect of the bootstrap size on the structure of the point cloud formed by the bootstrapped persistence images, we considered a population composed of a high number of traced microglia, namely the microglia of the adult, healthy dentate gyrus. This allowed us to span a range of sizes for the starting population from which we performed the bootstrapping. First, we only considered microglia traced from male animals to avoid inter-sex differences (**Fig. 1F**). As we observed no animal-specific batch effects (**Extended Data Fig. 1D**), we selected four mice with the highest number of tracings, and grouped them into two artificial groups (A and B) so that the number of single cells were similar in each group ($N_A = 223, N_B = 231$). Despite coming from the same brain region, these two groups have a non-zero TMD distance ($d = 10.64$) which we call the TMD intrinsic distance. This intrinsic distance arises due to small but accumulated variations in the persistence images. To account for the effect of unequal starting population sizes between the groups, we randomly selected $n = 200$ traced microglia from each group to form the starting population. We then drew single microglia from these groups to create a set of bootstrapped persistence images*,* which we call bootstrap samples A and B. Note that the TMD intrinsic distance remains the same, regardless of the bootstrap size (**Extended Data Fig. 2A**).

To characterize the point cloud formed by the bootstrapped persistence images, we calculated the within-condition distance which is the average TMD distance between two persistence images within the same bootstrap sample. We want to stress here that the two conditions A and B are artificial: the bootstrap persistence images in bootstrap samples A and B come from the same brain region. We observed that reducing the bootstrap size compacted the point cloud and subsequently reduced the within-condition distance within the bootstrap sample (**Extended Data Fig. 2C**). At a certain bootstrap-to-starting population size ratio where $N_A = N_B = 200$ the within-condition distance becomes smaller than the TMD intrinsic distance. This implies that the between-condition distances, *i.e.* the TMD distance between persistence images across different bootstrap samples, increases causing a forced separation between two groups from the same condition.

Thus, it is imperative to select a bootstrap size which reduces the dispersion of the bootstrap samples without artificially separating samples that share topological signatures. One way to address the latter condition is to determine whether the bootstrap samples A and B cluster

separately under a given bootstrap size. To test this, we performed a complete-linkage hierarchical clustering with the TMD distance as the metric. We imposed a cut-off which results in two clusters, $\omega_1$ and$\omega_2$. We then defined a mixing entropy of the resulting clusters $\Omega = \{\omega_1, \omega_2\}$ which measures the discrimination between bootstrap samples and is calculated as

$$H[\Omega] = \sum_{\omega=\{\omega_1,\omega_2\}} H[\omega]\frac{N_\omega}{N}$$

where $H[\omega] = \frac{N_A(\omega)}{N_\omega}\log_2\frac{N_A(\omega)}{N_\omega} - \left(1-\frac{N_A(\omega)}{N_\omega}\right)\log_2\left(1-\frac{N_A(\omega)}{N_\omega}\right)$ is the entropy of cluster$\omega$, $N_A(\omega)$ is the number of bootstrapped persistence images in bootstrap sample A located in cluster $\omega$ and $N = N_{\omega_1} + N_{\omega_2}$ is the total number of bootstrapped persistence images in the point cloud. To understand this mixing entropy, we considered both extreme situations. The first case is when bootstrap samples A and B overlap and the point cloud is highly dispersed: this results in a big cluster with all persistence images aggregated except for one. The latter is the last bootstrap persistence image to be clustered in the dendrogram, and the mixing entropy is close to 1. On the other hand, when the point cloud dispersion was small enough for bootstrap samples A and B to segregate, the clustering resulted in separate clusters for samples A and B, and the mixing entropy is zero. Note, the latter emerges whenever we force the separation between two similar-condition groups.

If we also consider the situation between the two extreme situations, the mixing entropy decreases with increasing bootstrap-to-starting population size ratio (**Extended Data Fig. 2C**). We observed that the mixing entropy remained close to 1 for a range of small ratios and then dropped to zero, depending on the starting population size. This behavior allows us to define an optimal bootstrap size which maximizes the trade-off between the intrinsic variability of the bootstrap samples and the indistinguishability of samples coming from the same conditions. By dividing the mixing entropy with the within-condition distance, we found a peak close to a bootstrap size that is 30% of the starting population size (**Extended Data Fig. 2D**).

Furthermore, we assessed the effect of low starting population sizes on both the within-condition distances and mixing entropy. Thus, we randomly selected $n = \{10, 20, 30, 40, 50, 60, 70, 80\}$ microglia from each group and performed the bootstrapping over the resulting starting populations. We found that both within-condition distance and

mixing entropy decreased as a function of the bootstrap size, but dependent on the starting population size (**Extended Data Fig. 2C**). Interestingly, when we divided the mixing entropy by the within-condition distance, we observed that there was no longer a well-defined optimal bootstrap size (**Extended Data Fig. 2D**), and that the optimal ratio is a range of parameters which is larger for samples with a low starting population size.

***2. Multiple-condition case: sexual dimorphism in the frontal cortex and dentate gyrus***

In the analyses above, we only considered the situation where two groups come from the same condition, such as microglia from the adult male dentate gyrus. Here, we look at a situation where we have multiple conditions which not only exhibit spatial but also sexual heterogeneity.

We focused on microglial populations coming from the healthy, adult frontal cortex (FC) and the dentate gyrus (DG) where we see a microglial signature and a region-dependent sexual dimorphism. Thus, we took 75 microglia from the four male and four female mice with the highest number of traced microglia in the frontal cortex and dentate gyrus. To investigate the effect of having unequal proportions of male and female microglia in the sample, we created starting populations with size $N = 200$ where the male-to-female microglia ratio, $r = \{0.0, 0.1, 0.2, \ldots, 0.8, 0.9, 1.0\}$ was fixed. For each brain region and male-to-female ratio, we constructed bootstrap samples with bootstrap-to-starting population size ratio at 0.1, 0.3, 0.5, 0.7, and 0.9. As there are multiple conditions, we looked at the 2D UMAP representations for each bootstrap size using the same parameters in the main text.

We observed that at 0.1 size ratio, the difference in the morphological signature between FC and DG microglia is already apparent (**Extended Data Fig. 2E**), and that within a brain region cluster, the pure male and female samples are located at opposite ends. These ends in the $DG_{mg}$ cluster tend to come closer together than those of the $FC_{mg}$. Note that these observations are captured when the bootstrap-to-starting population size ratio is at 0.3.

Finally, we observed that as the bootstrap size increased, samples with different male-to-female size ratios broke apart, suggesting a forced separation between different conditions. However, the rate at which the different conditions became more distinct was not uniform. Indeed, we saw that the male-dominated samples in the $DG_{mg}$ still form a cluster at bootstrap-to-starting population size ratio 0.9, which implies that ♂$DG_{mg}$ have "stronger" and less

variable microglial signatures than their female counterparts. This suggests that spanning a range of size ratios can uncover information on the intrinsic variability.

## Reference

1. Tremblay, M.-È. *et al.* The role of microglia in the healthy brain. *J. Neurosci.* **31**, 16064–9 (2011).

# Supplementary Table 1 – related to Extended Data Figure 1A

## Dendritic length

| | CB | CN | DG | FC | OB | S1 | SN |
|---|---|---|---|---|---|---|---|
| CB | 1.000E+00 | 1.000E+00 | 3.910E-06 | 8.771E-05 | 2.755E-07 | 4.132E-11 | 1.396E-04 |
| CN | 1.000E+00 | 1.000E+00 | 3.612E-04 | 4.258E-03 | 3.982E-05 | 1.706E-08 | 5.711E-03 |
| DG | 3.910E-06 | 3.612E-04 | 1.000E+00 | 1.000E+00 | 1.000E+00 | 7.192E-01 | 1.000E+00 |
| FC | 8.771E-05 | 4.258E-03 | 1.000E+00 | 1.000E+00 | 1.000E+00 | 2.127E-01 | 1.000E+00 |
| OB | 2.755E-07 | 3.982E-05 | 1.000E+00 | 1.000E+00 | 1.000E+00 | 1.000E+00 | 1.000E+00 |
| S1 | 4.132E-11 | 1.706E-08 | 7.192E-01 | 2.127E-01 | 1.000E+00 | 1.000E+00 | 2.595E-01 |
| SN | 1.396E-04 | 5.711E-03 | 1.000E+00 | 1.000E+00 | 1.000E+00 | 2.595E-01 | 1.000E+00 |

## Number of branches

| | CB | CN | DG | FC | OB | S1 | SN |
|---|---|---|---|---|---|---|---|
| CB | 1.000E+00 | 1.000E+00 | 1.533E-07 | 4.747E-05 | 4.124E-09 | 1.330E-10 | 2.383E-05 |
| CN | 1.000E+00 | 1.000E+00 | 2.932E-03 | 1.047E-01 | 2.475E-04 | 1.398E-05 | 5.860E-02 |
| DG | 1.533E-07 | 2.932E-03 | 1.000E+00 | 1.000E+00 | 1.000E+00 | 1.000E+00 | 1.000E+00 |
| FC | 4.747E-05 | 1.047E-01 | 1.000E+00 | 1.000E+00 | 1.000E+00 | 4.746E-01 | 1.000E+00 |
| OB | 4.124E-09 | 2.475E-04 | 1.000E+00 | 1.000E+00 | 1.000E+00 | 1.000E+00 | 1.000E+00 |
| S1 | 1.330E-10 | 1.398E-05 | 1.000E+00 | 4.746E-01 | 1.000E+00 | 1.000E+00 | 9.989E-01 |
| SN | 2.383E-05 | 5.860E-02 | 1.000E+00 | 1.000E+00 | 1.000E+00 | 9.989E-01 | 1.000E+00 |

## Number of branching points

| | CB | CN | DG | FC | OB | S1 | SN |
|---|---|---|---|---|---|---|---|
| CB | 1.000E+00 | 1.000E+00 | 2.334E-07 | 4.446E-05 | 4.897E-09 | 1.197E-10 | 2.250E-05 |
| CN | 1.000E+00 | 1.000E+00 | 4.192E-03 | 1.066E-01 | 3.044E-04 | 1.424E-05 | 6.001E-02 |
| DG | 2.334E-07 | 4.192E-03 | 1.000E+00 | 1.000E+00 | 1.000E+00 | 1.000E+00 | 1.000E+00 |
| FC | 4.446E-05 | 1.066E-01 | 1.000E+00 | 1.000E+00 | 1.000E+00 | 4.721E-01 | 1.000E+00 |
| OB | 4.897E-09 | 3.044E-04 | 1.000E+00 | 1.000E+00 | 1.000E+00 | 1.000E+00 | 1.000E+00 |
| S1 | 1.197E-10 | 1.424E-05 | 1.000E+00 | 4.721E-01 | 1.000E+00 | 1.000E+00 | 9.904E-01 |
| SN | 2.250E-05 | 6.001E-02 | 1.000E+00 | 1.000E+00 | 1.000E+00 | 9.904E-01 | 1.000E+00 |

## Number of terminal points

| | CB | CN | DG | FC | OB | S1 | SN |
|---|---|---|---|---|---|---|---|
| CB | 1.000E+00 | 1.000E+00 | 9.996E-08 | 4.816E-05 | 3.461E-09 | 1.479E-10 | 2.669E-05 |
| CN | 1.000E+00 | 1.000E+00 | 2.263E-03 | 1.079E-01 | 2.262E-04 | 1.563E-05 | 6.440E-02 |
| DG | 9.996E-08 | 2.263E-03 | 1.000E+00 | 1.000E+00 | 1.000E+00 | 1.000E+00 | 1.000E+00 |
| FC | 4.816E-05 | 1.079E-01 | 1.000E+00 | 1.000E+00 | 1.000E+00 | 4.903E-01 | 1.000E+00 |
| OB | 3.461E-09 | 2.262E-04 | 1.000E+00 | 1.000E+00 | 1.000E+00 | 1.000E+00 | 1.000E+00 |
| S1 | 1.479E-10 | 1.563E-05 | 1.000E+00 | 4.903E-01 | 1.000E+00 | 1.000E+00 | 9.820E-01 |
| SN | 2.669E-05 | 6.440E-02 | 1.000E+00 | 1.000E+00 | 1.000E+00 | 9.820E-01 | 1.000E+00 |

**Supplementary Table 1. Statistical tests related to Extended Data Fig. 1A**

*P*-value for pairwise comparisons between adult microglia of a brain regions for each morphometric. Cerebellum (CB), cochlear nucleus (CN), dentate gyrus (DG), frontal cortex (FC), olfactory bulb (OB), somatosensory cortex (S1), substantia nigra (SN).

# Supplementary Table 2 – related to Extended Data Figure 10A

**Process length**

| | | ♂ | | | | ♀ | | | |
|---|---|---|---|---|---|---|---|---|---|
| | | Control | 1 week | 2 weeks | 6 weeks | Control | 1 week | 2 weeks | 6 weeks |
| ♂ | Control | 1.00E+00 | 1.83E-17 | 1.28E-70 | 3.80E-131 | 1.00E+00 | 1.00E+00 | 8.40E-22 | 4.95E-85 |
| | 1 week | 1.83E-17 | 1.00E+00 | 5.13E-03 | 1.27E-15 | 7.59E-23 | 1.90E-08 | 1.00E+00 | 4.46E-02 |
| | 2 weeks | 1.28E-70 | 5.13E-03 | 1.00E+00 | 2.55E-08 | 6.97E-86 | 4.71E-28 | 1.29E-03 | 1.00E+00 |
| | 6 weeks | 3.80E-131 | 1.27E-15 | 2.55E-08 | 1.00E+00 | 1.40E-151 | 3.74E-56 | 1.34E-18 | 3.16E-14 |
| ♀ | Control | 1.00E+00 | 7.59E-23 | 6.97E-86 | 1.40E-151 | 1.00E+00 | 3.39E-01 | 1.57E-28 | 4.04E-105 |
| | 1 week | 1.00E+00 | 1.90E-08 | 4.71E-28 | 3.74E-56 | 3.39E-01 | 1.00E+00 | 1.01E-09 | 8.04E-28 |
| | 2 weeks | 8.40E-22 | 1.00E+00 | 1.29E-03 | 1.34E-18 | 1.57E-28 | 1.01E-09 | 1.00E+00 | 1.39E-02 |
| | 6 weeks | 4.95E-85 | 4.46E-02 | 1.00E+00 | 3.16E-14 | 4.04E-105 | 8.04E-28 | 1.39E-02 | 1.00E+00 |

**Number of branches**

| | | ♂ | | | | ♀ | | | |
|---|---|---|---|---|---|---|---|---|---|
| | | Control | 1 week | 2 weeks | 6 weeks | Control | 1 week | 2 weeks | 6 weeks |
| ♂ | Control | 1.00E+00 | 1.83E-17 | 1.28E-70 | 3.80E-131 | 1.00E+00 | 1.00E+00 | 8.40E-22 | 4.95E-85 |
| | 1 week | 1.83E-17 | 1.00E+00 | 5.13E-03 | 1.27E-15 | 7.59E-23 | 1.90E-08 | 1.00E+00 | 4.46E-02 |
| | 2 weeks | 1.28E-70 | 5.13E-03 | 1.00E+00 | 2.55E-08 | 6.97E-86 | 4.71E-28 | 1.29E-03 | 1.00E+00 |
| | 6 weeks | 3.80E-131 | 1.27E-15 | 2.55E-08 | 1.00E+00 | 1.40E-151 | 3.74E-56 | 1.34E-18 | 3.16E-14 |
| ♀ | Control | 1.00E+00 | 7.59E-23 | 6.97E-86 | 1.40E-151 | 1.00E+00 | 3.39E-01 | 1.57E-28 | 4.04E-105 |
| | 1 week | 1.00E+00 | 1.90E-08 | 4.71E-28 | 3.74E-56 | 3.39E-01 | 1.00E+00 | 1.01E-09 | 8.04E-28 |
| | 2 weeks | 8.40E-22 | 1.00E+00 | 1.29E-03 | 1.34E-18 | 1.57E-28 | 1.01E-09 | 1.00E+00 | 1.39E-02 |
| | 6 weeks | 4.95E-85 | 4.46E-02 | 1.00E+00 | 3.16E-14 | 4.04E-105 | 8.04E-28 | 1.39E-02 | 1.00E+00 |

**Number of branching points**

| | | ♂ | | | | ♀ | | | |
|---|---|---|---|---|---|---|---|---|---|
| | | Control | 1 week | 2 weeks | 6 weeks | Control | 1 week | 2 weeks | 6 weeks |
| ♂ | Control | 1.00E+00 | 9.96E-27 | 3.61E-100 | 1.32E-102 | 1.00E+00 | 3.66E-01 | 7.20E-24 | 1.00E-60 |
| | 1 week | 9.96E-27 | 1.00E+00 | 1.31E-03 | 1.80E-04 | 3.13E-32 | 1.71E-10 | 1.00E+00 | 1.00E+00 |
| | 2 weeks | 3.61E-100 | 1.31E-03 | 1.00E+00 | 1.00E+00 | 1.57E-115 | 3.17E-34 | 1.75E-08 | 2.01E-11 |
| | 6 weeks | 1.32E-102 | 1.80E-04 | 1.00E+00 | 1.00E+00 | 8.55E-118 | 2.92E-36 | 8.83E-10 | 2.97E-13 |
| ♀ | Control | 1.00E+00 | 3.13E-32 | 1.57E-115 | 8.55E-118 | 1.00E+00 | 9.41E-03 | 9.93E-30 | 5.78E-75 |
| | 1 week | 3.66E-01 | 1.71E-10 | 3.17E-34 | 2.92E-36 | 9.41E-03 | 1.00E+00 | 9.21E-08 | 3.02E-14 |
| | 2 weeks | 7.20E-24 | 1.00E+00 | 1.75E-08 | 8.83E-10 | 9.93E-30 | 9.21E-08 | 1.00E+00 | 1.00E+00 |
| | 6 weeks | 1.00E-60 | 1.00E+00 | 2.01E-11 | 2.97E-13 | 5.78E-75 | 3.02E-14 | 1.00E+00 | 1.00E+00 |

**Number of terminal points**

| | | ♂ | | | | ♀ | | | |
|---|---|---|---|---|---|---|---|---|---|
| | | Control | 1 week | 2 weeks | 6 weeks | Control | 1 week | 2 weeks | 6 weeks |
| ♂ | Control | 1.00E+00 | 2.26E-27 | 7.97E-102 | 5.11E-100 | 1.00E+00 | 2.90E-01 | 1.68E-23 | 2.25E-60 |
| | 1 week | 2.26E-27 | 1.00E+00 | 1.34E-03 | 7.40E-04 | 7.39E-33 | 1.26E-10 | 1.00E+00 | 1.00E+00 |
| | 2 weeks | 7.97E-102 | 1.34E-03 | 1.00E+00 | 1.00E+00 | 4.22E-117 | 1.77E-34 | 4.68E-09 | 4.03E-12 |
| | 6 weeks | 5.11E-100 | 7.40E-04 | 1.00E+00 | 1.00E+00 | 8.48E-115 | 8.97E-35 | 2.13E-09 | 1.82E-12 |
| ♀ | Control | 1.00E+00 | 7.39E-33 | 4.22E-117 | 8.48E-115 | 1.00E+00 | 7.28E-03 | 3.13E-29 | 2.27E-74 |
| | 1 week | 2.90E-01 | 1.26E-10 | 1.77E-34 | 8.97E-35 | 7.28E-03 | 1.00E+00 | 2.01E-07 | 7.46E-14 |
| | 2 weeks | 1.68E-23 | 1.00E+00 | 4.68E-09 | 2.13E-09 | 3.13E-29 | 2.01E-07 | 1.00E+00 | 1.00E+00 |
| | 6 weeks | 2.25E-60 | 1.00E+00 | 4.03E-12 | 1.82E-12 | 2.27E-74 | 7.46E-14 | 1.00E+00 | 1.00E+00 |

**Supplementary Table 2. Statistical tests related to Extended Data Fig. 10A**

*P*-value for pairwise comparison between microglia from the frontal cortex of the CK-p25 for different conditions and sex.

## Supplementary Table 3 –
## related to Figure 6A and Extended Data Fig. 10B-C

### EXTENDED LIST OF MORPHOMETRIC QUANTITIES

| | L-measure metric | |
|---|---|---|
| **1. Whole tree/microglia size** | | |
| Summed total process length | *Length* | *Total_Sum* |
| Number of process tips | *N_tips* | *Total_Sum* |
| Total process width | *Width* | *Total_Sum* |
| Total process height | *Height* | *Total_Sum* |
| Total process depth | *Depth* | *Total_Sum* |
| **2. Bifurcation measures** | | |
| Average partition asymmetry | *Partition_Asymmetry* | *Average* |
| Average local amplitude angle | *Bif_ampl_local* | *Average* |
| Maximum local amplitude angle | *Bif_ampl_local* | *Maximum* |
| Average remote amplitude angle | *Bif_ampl_remote* | *Average* |
| Maximum remote amplitude angle | *Bif_ampl_remote* | *Maximum* |
| Average local tilt angle | *Bif_tilt_local* | *Average* |
| Maximum local tilt angle | *Bif_tilt_local* | *Maximum* |
| Average remote tilt angle | *Bif_tilt_remote* | *Average* |
| Maximum remote tilt angle | *Bif_tilt_remote* | *Maximum* |
| Average local torque angle | *Bif_torque_local* | *Average* |
| Maximum local torque angle | *Bif_torque_local* | *Maximum* |
| Average remote torque angle | *Bif_torque_remote* | *Average* |
| Maximum remote torque angle | *Bif_torque_remote* | *Maximum* |
| **3. Process measures** | | |
| Average tortuosity | *Contraction* | *Average* |
| Average fractal dimension | *Fractal_Dim* | *Average* |
| Maximum fractal dimension | *Fractal_Dim* | *Maximum* |
| Average branch path length | *Branch_pathlength* | *Average* |
| Maximum branch path length | *Branch_pathlength* | *Maximum* |
| **4. Compartment measures** | | |
| Maximum branch order | *Branch_Order* | *Maximum* |
| Average terminal degree | *Terminal_degree* | *Average* |
| Maximum path distance from soma | *PathDistance* | *Maximum* |
| Maximum branch helicity | *Helix* | *Maximum* |

**Supplementary Table 3. Classical morphometric related to Figure 6A, Extended Data Fig. 10B-C.**

Extended list of classical morphometric quantities [51,81].

# Supplementary Table 4

### Cerebellum (CB)

| Condition | Time point | Total (excl.) | ♂ | ♀ | ♀ov |
|---|---|---|---|---|---|
| Adult | 8-12 weeks | 607 (2) | 246 | 299 | 60 |
| Development | P7 | 399 (0) | 189 | 210 | |
| | P15 | 685 (0) | 175 | 510 | |
| | P22 | 656 (4) | 440 | 212 | |
| 5xFAD | 3 months | 111 (2) | 62 | 47 | |
| | 6 months | 139 (1) | 72 | 66 | |
| CK-p25 | 1 week | 122 (0) | 71 | 51 | |
| | 2 weeks | 66 (1) | 33 | 32 | |
| | 6 weeks | 213 (1) | 97 | 115 | |

### Cochlear nucleus (CN)

| Condition | Time point | Total | ♂ | ♀ | ♀ov |
|---|---|---|---|---|---|
| Adult | 8-12 weeks | 831 (0) | 256 | 498 | 77 |
| Development | P7 | 478 (1) | 252 | 225 | |
| | P15 | 747 (0) | 413 | 334 | |
| | P22 | 659 (21) | 311 | 327 | |
| 5xFAD | 3 months | 236 (0) | 117 | 119 | |
| | 6 months | 170 (0) | 67 | 103 | |
| CK-p25 | 1 week | 208 (0) | 129 | 79 | |
| | 2 weeks | 249 (1) | 133 | 115 | |
| | 6 weeks | 209 (0) | 72 | 137 | |

### Frontal cortex (FC)

| Condition | Time point | Total (excl.) | ♂ | ♀ | ♀ov |
|---|---|---|---|---|---|
| Adult | 8-12 weeks | 2014(1) | 894 | 926 | 193 |
| Development | P7 | 378 (1) | 184 | 193 | |
| | P15 | 953 (0) | 584 | 369 | |
| | P22 | 877 (0) | 407 | 470 | |
| 5xFAD | 3 months | 355 (0) | 250 | 105 | |
| | 6 months | 443 (0) | 180 | 263 | |
| CK-p25 | 1 week | 413 (0) | 194 | 219 | |
| | 2 weeks | 756 (0) | 492 | 264 | |
| | 6 weeks | 1321(1) | 462 | 858 | |

### Dentate gyrus (DG)

| Condition | Time point | Total | ♂ | ♀ | ♀ov |
|---|---|---|---|---|---|
| Adult | 8-12 weeks | 1929(1) | 913 | 902 | 116 |
| Development | P7 | 368 (1) | 154 | 214 | |
| | P15 | 439 (0) | 289 | 150 | |
| | P22 | 665 (0) | 286 | 379 | |
| 5xFAD | 3 months | 643 (0) | 216 | 427 | |
| | 6 months | 533 (0) | 303 | 230 | |
| CK-p25 | 1 week | 383 (0) | 223 | 160 | |
| | 2 weeks | 593 (0) | 307 | 286 | |
| | 6 weeks | 1630 (1) | 293 | 1336 | |

### Olfactory bulb (OB)

| Condition | Time point | Total (excl.) | ♂ | ♀ | ♀ov |
|---|---|---|---|---|---|
| Adult | 8-12 weeks | 1671(1) | 701 | 796 | 173 |
| Development | P7 | 274 (3) | 196 | 75 | |
| | P15 | 477 (0) | 309 | 168 | |
| | P22 | 698 (0) | 390 | 308 | |
| 5xFAD | 3 months | 285 (0) | 82 | 203 | |
| | 6 months | 776 (0) | 244 | 527 | |
| CK-p25 | 1 week | 285 (0) | 144 | 140 | |
| | 2 weeks | 657 (0) | 223 | 432 | |
| | 6 weeks | 640 (0) | 235 | 405 | |

### Somatosensory cortex (S1)

| Condition | Time point | Total | ♂ | ♀ | ♀ov |
|---|---|---|---|---|---|
| Adult | 8-12 weeks | 1710(3) | 821 | 719 | 167 |
| Development | P7 | 312 (0) | 165 | 147 | |
| | P15 | 688 (0) | 458 | 230 | |
| | P22 | 786 (0) | 506 | 280 | |
| 5xFAD | 3 months | 540 (0) | 254 | 286 | |
| | 6 months | 629 (1) | 374 | 254 | |
| CK-p25 | 1 week | 443 (2) | 177 | 264 | |
| | 2 weeks | 479 (0) | 273 | 206 | |
| | 6 weeks | 835 (1) | 231 | 603 | |
| KXA | 1x KXA | 377 (4) | 213 | 160 | |
| | 2x KXA | 295 (0) | 159 | 136 | |
| | 3x KXA | 292 (2) | 136 | 154 | |
| Recovery after 3x KXA | 3 days | 223 (0) | 115 | 108 | |
| | 1-week | 258 (2) | 123 | 133 | |
| | 2-weeks | 297 (0) | 171 | 126 | |

### Substantia nigra (SN)

| Condition | Time point | Total (excl.) | ♂ | ♀ | ♀ov |
|---|---|---|---|---|---|
| Adult | 8-12 weeks | 2264(2) | 976 | 1050 | 236 |
| Development | P7 | 549 (2) | 350 | 197 | |
| | P15 | 943 (0) | 405 | 538 | |
| | P22 | 644 (0) | 312 | 332 | |
| 5xFAD | 3 months | 319 (0) | 166 | 153 | |
| | 6 months | 460 (0) | 202 | 258 | |
| CK-p25 | 1 week | 265 (1) | 116 | 148 | |
| | 2 weeks | 450 (1) | 249 | 200 | |
| | 6 weeks | 717 (1) | 469 | 247 | |

**Supplementary Table 4. Number of traced microglia for each brain region, sex, and condition**

Total number of traced cells. In brackets, number of excluded cells that did not pass the quality check and were discarded from further analysis. $♀_{ov}$, ovariectomized females.

# Supplementary Table 5

| Region | Condition | Time point | ♂ | ♀ | ♀ov |
|---|---|---|---|---|---|
| Cerebellum (CB) | Adult | 8-12 weeks | 12 | 9 | 4 |
| | Develop-ment | P7 | 3 | 3 | |
| | | P15 | 3 | 3 | |
| | | P22 | 3 | 3 | |
| | 5xFAD | 3 months | 3 | 3 | |
| | | 6 months | 3 | 3 | |
| | CK-p25 | 1 week | 3 | 3 | |
| | | 2 weeks | 2 | 3 | |
| | | 6 weeks | 3 | 3 | |

| Region | Condition | Time point | ♂ | ♀ | ♀ov |
|---|---|---|---|---|---|
| Cochlear nucleus (CN) | Adult | 8-12 weeks | 8 | 12 | 4 |
| | Develop-ment | P7 | 3 | 3 | |
| | | P15 | 3 | 3 | |
| | | P22 | 3 | 3 | |
| | 5xFAD | 3 months | 3 | 3 | |
| | | 6 months | 3 | 3 | |
| | CK-p25 | 1 week | 3 | 3 | |
| | | 2 weeks | 4 | 3 | |
| | | 6 weeks | 3 | 3 | |

| Region | Condition | Time point | ♂ | ♀ | ♀ov |
|---|---|---|---|---|---|
| Frontal cortex (FC) | Adult | 8-12 weeks | 12 | 12 | 4 |
| | Develop-ment | P7 | 3 | 3 | |
| | | P15 | 3 | 3 | |
| | | P22 | 3 | 3 | |
| | 5xFAD | 3 months | 3 | 2 | |
| | | 6 months | 2 | 3 | |
| | CK-p25 | 1 week | 2 | 3 | |
| | | 2 weeks | 5 | 3 | |
| | | 6 weeks | 4 | 5 | |

| Region | Condition | Time point | ♂ | ♀ | ♀ov |
|---|---|---|---|---|---|
| Dentate gyrus (DG) | Adult | 8-12 weeks | 11 | 11 | 4 |
| | Develop-ment | P7 | 3 | 3 | |
| | | P15 | 3 | 3 | |
| | | P22 | 3 | 3 | |
| | 5xFAD | 3 months | 3 | 3 | |
| | | 6 months | 3 | 3 | |
| | CK-p25 | 1 week | 4 | 3 | |
| | | 2 weeks | 3 | 3 | |
| | | 6 weeks | 5 | 7 | |

| Region | Condition | Time point | ♂ | ♀ | ♀ov |
|---|---|---|---|---|---|
| Olfactory bulb (OB) | Adult | 8-12 weeks | 12 | 12 | 4 |
| | Develop-ment | P7 | 3 | 3 | |
| | | P15 | 3 | 3 | |
| | | P22 | 3 | 3 | |
| | 5xFAD | 3 months | 2 | 4 | |
| | | 6 months | 3 | 4 | |
| | CK-p25 | 1 week | 2 | 2 | |
| | | 2 weeks | 4 | 4 | |
| | | 6 weeks | 3 | 3 | |

| Region | Condition | Time point | ♂ | ♀ | ♀ov |
|---|---|---|---|---|---|
| Somatosensory cortex (S1) | Adult | 8-12 weeks | 10 | 10 | 4 |
| | Develop-ment | P7 | 3 | 3 | |
| | | P15 | 3 | 3 | |
| | | P22 | 3 | 3 | |
| | 5xFAD | 3 months | 4 | 3 | |
| | | 6 months | 3 | 3 | |
| | CK-p25 | 1 week | 3 | 3 | |
| | | 2 weeks | 3 | 3 | |
| | | 6 weeks | 5 | 4 | |
| | KXA | 1x KXA | 3 | 3 | |
| | | 2x KXA | 3 | 3 | |
| | | 3x KXA | 3 | 3 | |
| | Recovery after 3x KXA | 3 days | 3 | 3 | |
| | | 1-week | 3 | 3 | |
| | | 2-weeks | 3 | 3 | |

| Region | Condition | Time point | ♂ | ♀ | ♀ov |
|---|---|---|---|---|---|
| Substantia nigra (SN) | Adult | 8-12 weeks | 10 | 10 | 4 |
| | Develop-ment | P7 | 3 | 3 | |
| | | P15 | 3 | 3 | |
| | | P22 | 4 | 2 | |
| | 5xFAD | 3 months | 3 | 3 | |
| | | 6 months | 3 | 3 | |
| | CK-p25 | 1 week | 3 | 3 | |
| | | 2 weeks | 4 | 3 | |
| | | 6 weeks | 3 | 4 | |

**Supplementary Table 5. Number of animals used**

Total number of animals used per condition. ♀ov, ovariectomized females.
