# Supplementary material for: A tool for mapping microglial morphology, morphOMICs, reveals brain-region and sex-dependent phenotypes
Source: Nat Neurosci. 2022 Sep 30;25(10):1379–93. doi: 10.1038/s41593-022-01167-6 (PMC9534764; doi:10.1038/s41593-022-01167-6)
Supplement: Supplementary file 2 — Reporting Summary [file 41593_2022_1167_MOESM2_ESM.pdf]

nature portfolio

Corresponding author(s): Sandra Siegert

Last updated by author(s): Aug 5, 2022

Reporting Summary

Nature Portfolio wishes to improve the reproducibility of the work that we publish. This form provides structure for consistency and transparency in reporting. For further information on Nature Portfolio policies, see our Editorial Policies and the Editorial Policy Checklist.

Statistics

For all statistical analyses, confirm that the following items are present in the figure legend, table legend, main text, or Methods section.

n/a	Confirmed	
☐	☒	The exact sample size (*n*) for each experimental group/condition, given as a discrete number and unit of measurement
☐	☒	A statement on whether measurements were taken from distinct samples or whether the same sample was measured repeatedly
☐	☒	The statistical test(s) used AND whether they are one- or two-sided<br>*Only common tests should be described solely by name; describe more complex techniques in the Methods section.*
☐	☒	A description of all covariates tested
☐	☒	A description of any assumptions or corrections, such as tests of normality and adjustment for multiple comparisons
☐	☒	A full description of the statistical parameters including central tendency (e.g. means) or other basic estimates (e.g. regression coefficient) AND variation (e.g. standard deviation) or associated estimates of uncertainty (e.g. confidence intervals)
☐	☒	For null hypothesis testing, the test statistic (e.g. *F*, *t*, *r*) with confidence intervals, effect sizes, degrees of freedom and *P* value noted<br>*Give P values as exact values whenever suitable.*
☒	☐	For Bayesian analysis, information on the choice of priors and Markov chain Monte Carlo settings
☒	☐	For hierarchical and complex designs, identification of the appropriate level for tests and full reporting of outcomes
☒	☐	Estimates of effect sizes (e.g. Cohen's *d*, Pearson's *r*), indicating how they were calculated

Our web collection on statistics for biologists contains articles on many of the points above.

Software and code

Policy information about availability of computer code

Data collection	Details about obtaining 3D-microglial morphologies are outlined in the Material and Methods under the sections: Animals, Brain samples and analyzed brain regions, Ovariectomy, Transcardiac perfusion, Vibratome sections, Immunofluorescence staining, Confocal microscopy, Image processing, and Reconstruction of 3D-traced microglia.<br><br>For data collection, the following codes/softwares were used: Imaris Stitcher v9.3.1, Fiji 1.52e, in Imaris 9.2.v (Bitplane Imaris), ImarisReader toolbox for MATLAB (https://github.com/PeterBeemiller/ImarisReader), NL Morphology Converter (http://neuroland.org).
Data analysis	Data analysis is described in Material and Methods sections of the manuscript under the sections: Analysis of morphometric features, Topological morphology descriptor (TMD), Average and bootstrapped persistence images, TMD distance, Hierarchical clustering, Dimensionality reduction, tSNE, Pseudotemporal ordering, Palantir, Monocle, Stable ranks analysis, and Bootstrapped morphometric features. All the MorphOMICs codes used are available through a GitHub library: https://git.ist.ac.at/rcubero/morphomics.<br><br>On top of the MorphOMICs codes, the following codes/softwares were used: L-measure (http://cng.gmu.edu:8080/Lm/), NeuroM Python toolkit (https://github.com/BlueBrain/NeuroM), cluster.hierarchy.linkage from SciPy v1.6.2, sklearn.svm.SVC from sklearn (https://scikit-learn.org/stable/), scipy.stats (v1.6.2) and scikit-posthocs (v0.6.7) and R (v3.4.4).

For manuscripts utilizing custom algorithms or software that are central to the research but not yet described in published literature, software must be made available to editors and reviewers. We strongly encourage code deposition in a community repository (e.g. GitHub). See the Nature Portfolio guidelines for submitting code & software for further information.

Data

Policy information about availability of data

All manuscripts must include a data availability statement. This statement should provide the following information, where applicable:

- Accession codes, unique identifiers, or web links for publicly available datasets
- A description of any restrictions on data availability
- For clinical datasets or third party data, please ensure that the statement adheres to our policy

All the .swc files are available at the NeuroMorpho.org repository in the Siegert archive under the link: https://neuromorpho.org/KeywordResult.jsp?keywords=%22siegert%22.

We have also used the Allen Developing Mouse Brain Atlas - Sagittal atlas to illustrate the locations of brain regions-of-interest. The image was taken from https://atlas.brain-map.org/atlas?atlas=2#atlas=2&plate=100883770&structure=549&x=7799.7333984375&y=4022.93359375&zoom=-3&resolution=16.75&z=5.

Field-specific reporting

Please select the one below that is the best fit for your research. If you are not sure, read the appropriate sections before making your selection.

☒ Life sciences ☐ Behavioural & social sciences ☐ Ecological, evolutionary & environmental sciences

For a reference copy of the document with all sections, see nature.com/documents/nr-reporting-summary-flat.pdf

Life sciences study design

All studies must disclose on these points even when the disclosure is negative.

Sample size	No statistical methods were used to pre-determine sample sizes but our sample sizes are similar to those reported in previous publications (Morrison et. al., 2013, Heindl et. al., 2018, Kongsui et. al., 2014, Tan et. al., 2019, Stratoulias et. al., 2019, Bachstetter et. al., 2015, Salamanca et. al.,2019, Del Mar Fernandez-Arjona, et. al., 2017).
Data exclusions	1.) After the tracing, we manually removed cells that were sitting at the border of the image and were only partially traced so that these cells would not be analyzed.<br>2.) Morphologies with only one branch (and consequently, one topological bar) were excluded from the analysis.<br>No other exclusion criteria were imposed.
Replication	For each condition [C57BL/6J adult mice (8-12 weeks (=adult), ovariectomized); Cx3cr1GFP/- mice at postnatal time points P7, P15, P21; 5xFAD mice after 3 and 6 months; and CK-p25 mice 1, 2, and 6 weeks after doxycycline withdrawal], we analyzed seven brain regions [OB, FC, S1, DG, SN, CN, CB].<br><br>Each condition was analyzed for both sexes [male/female] with at least biological triplicates. A breakdown of animals used per condition is detailed in Extended Data Table 5.<br>The total number of traced microglia per condition divided by sex is shown in Extended Data Table 1.<br>All microglia reconstructed across animals (after exclusion condition, see above) were pooled together. Within each condition, no animal-specific differences in morphologies were detected (Extended Data Figure 1E), indicating successful replication.
Randomization	For every experimental group (as determined using the genotype and age of the mouse), we randomly selected the same number of female and male mice based on the mouse IDs, across different litters.<br>All reconstructed microglia morphologies (after imposing exclusion criteria) were pooled together. In the MorphOMICs implementation, boostrapped samples were obtained from random selection of microglia conditioned on group labels, which provides further data randomization.
Blinding	For the analysis, we pre-selected the brain regions to be analyzed in this study (see section “Brain samples and analyzed brain regions”). For each brain region, we obtained a 2x2 tile covering an approximate area of 0.1 mm^2 and kept the position consistent between animals. Over 330 images were collected from three independent scientists over the course of three years. The data collection of microglia was performed with a semi-automated reconstruction over this tile eliminating the need of a blinded sampling approach. All microglia within the tile were 3D-reconstructed. Microglia that were sitting at the border of the image and were only partially traced were excluded from the data analysis. No other exclusion has been performed. The dataset was then converted into individual skeletons that were provided to a non-biologist, who performed the data analysis based on the group labels but without knowing the meaning of the labels. Further blinding would not be feasible as the overall phenotypic differences of the brain during development and disease prevent efficient blinding.

Behavioural & social sciences study design

All studies must disclose on these points even when the disclosure is negative.

Study description	*Briefly describe the study type including whether data are quantitative, qualitative, or mixed-methods (e.g. qualitative cross-sectional, quantitative experimental, mixed-methods case study).*

Research sample	*State the research sample (e.g. Harvard university undergraduates, villagers in rural India) and provide relevant demographic information (e.g. age, sex) and indicate whether the sample is representative. Provide a rationale for the study sample chosen. For studies involving existing datasets, please describe the dataset and source.*
Sampling strategy	*Describe the sampling procedure (e.g. random, snowball, stratified, convenience). Describe the statistical methods that were used to predetermine sample size OR if no sample-size calculation was performed, describe how sample sizes were chosen and provide a rationale for why these sample sizes are sufficient. For qualitative data, please indicate whether data saturation was considered, and what criteria were used to decide that no further sampling was needed.*
Data collection	*Provide details about the data collection procedure, including the instruments or devices used to record the data (e.g. pen and paper, computer, eye tracker, video or audio equipment) whether anyone was present besides the participant(s) and the researcher, and whether the researcher was blind to experimental condition and/or the study hypothesis during data collection.*
Timing	*Indicate the start and stop dates of data collection. If there is a gap between collection periods, state the dates for each sample cohort.*
Data exclusions	*If no data were excluded from the analyses, state so OR if data were excluded, provide the exact number of exclusions and the rationale behind them, indicating whether exclusion criteria were pre-established.*
Non-participation	*State how many participants dropped out/declined participation and the reason(s) given OR provide response rate OR state that no participants dropped out/declined participation.*
Randomization	*If participants were not allocated into experimental groups, state so OR describe how participants were allocated to groups, and if allocation was not random, describe how covariates were controlled.*

Ecological, evolutionary & environmental sciences study design

All studies must disclose on these points even when the disclosure is negative.

Study description	*Briefly describe the study. For quantitative data include treatment factors and interactions, design structure (e.g. factorial, nested, hierarchical), nature and number of experimental units and replicates.*
Research sample	*Describe the research sample (e.g. a group of tagged Passer domesticus, all Stenocereus thurberi within Organ Pipe Cactus National Monument), and provide a rationale for the sample choice. When relevant, describe the organism taxa, source, sex, age range and any manipulations. State what population the sample is meant to represent when applicable. For studies involving existing datasets, describe the data and its source.*
Sampling strategy	*Note the sampling procedure. Describe the statistical methods that were used to predetermine sample size OR if no sample-size calculation was performed, describe how sample sizes were chosen and provide a rationale for why these sample sizes are sufficient.*
Data collection	*Describe the data collection procedure, including who recorded the data and how.*
Timing and spatial scale	*Indicate the start and stop dates of data collection, noting the frequency and periodicity of sampling and providing a rationale for these choices. If there is a gap between collection periods, state the dates for each sample cohort. Specify the spatial scale from which the data are taken*
Data exclusions	*If no data were excluded from the analyses, state so OR if data were excluded, describe the exclusions and the rationale behind them, indicating whether exclusion criteria were pre-established.*
Reproducibility	*Describe the measures taken to verify the reproducibility of experimental findings. For each experiment, note whether any attempts to repeat the experiment failed OR state that all attempts to repeat the experiment were successful.*
Randomization	*Describe how samples/organisms/participants were allocated into groups. If allocation was not random, describe how covariates were controlled. If this is not relevant to your study, explain why.*
Blinding	*Describe the extent of blinding used during data acquisition and analysis. If blinding was not possible, describe why OR explain why blinding was not relevant to your study.*

Did the study involve field work? ☐ Yes ☐ No

Field work, collection and transport

Field conditions	*Describe the study conditions for field work, providing relevant parameters (e.g. temperature, rainfall).*
Location	*State the location of the sampling or experiment, providing relevant parameters (e.g. latitude and longitude, elevation, water depth).*
Access & import/export	*Describe the efforts you have made to access habitats and to collect and import/export your samples in a responsible manner and in compliance with local, national and international laws, noting any permits that were obtained (give the name of the issuing authority, the date of issue, and any identifying information).*
Disturbance	*Describe any disturbance caused by the study and how it was minimized.*

Reporting for specific materials, systems and methods

We require information from authors about some types of materials, experimental systems and methods used in many studies. Here, indicate whether each material, system or method listed is relevant to your study. If you are not sure if a list item applies to your research, read the appropriate section before selecting a response.

Materials & experimental systems

n/a	Involved in the study
☐	☒ Antibodies
☒	☐ Eukaryotic cell lines
☒	☐ Palaeontology and archaeology
☐	☒ Animals and other organisms
☒	☐ Human research participants
☒	☐ Clinical data
☒	☐ Dual use research of concern

Methods

n/a	Involved in the study
☒	☐ ChIP-seq
☒	☐ Flow cytometry
☒	☐ MRI-based neuroimaging

Antibodies

Antibodies used

The following primary antibodies were used: rat α-CD68 (AbD Serotec, Cat#MCA1957, clone FA-11, Lot 1807); goat α-Iba1 (Abcam, ab5076, Lot FR3288145-1); rabbit anti-Iba1 (GeneTex, Cat#GTX100042, Lot 41556).
The secondary antibodies raised in goat or donkey were purchased from Thermo Fisher Scientific (Alexa Fluor 488 goat anti-rabbit IgG #A11034, Alexa Fluor 647 goat anti-rat IgG #21247, 1:2000).

Validation

All listed antibodies are commercially available. The same antibodies and Lot have been successfully used in a recently peer-reviewed study (Venturino et al., Cell Rep. 2021 Jul 6;36(1):109313. doi: 10.1016/j.celrep.2021.109313).

Animals and other organisms

Policy information about studies involving animals; ARRIVE guidelines recommended for reporting animal research

Laboratory animals

IST Austria: C57BL/6J (Cat#000664) and B6.129P-Cx3cr1tm1Litt/J (Cat#005582, named here Cx3cr1GFP/-, only heterozygous mice were used) were purchased from The Jackson Laboratories.
MIT: 5xFAD and CK-p25 mice were obtained from the Tsai lab at MIT. Tg(APPSwFlLon,PSEN1*M146L*L286V)6799Vas/Mmjax, Stock No: 34840-JAX) were obtained from Jackson laboratory. CK-p25 mice were generated by breeding CaMKIIα promoter-tTA mice (CK controls) (B6;CBA-Tg(Camk2a-tTA)1Mmay/J, Jackson Laboratory, Stock No: 003010) with tetO-CDK5R1/GFP mice (C57BL/6-Tg(tetO-CDK5R1/GFP)337Lht/J, Jackson Laboratory, Stock No: 005706). CK-p25 mice and their CK control littermates were conceived and raised in the presence of doxycycline-containing food to repress p25 transgene expression. To induce p25 transgene expression, mice were fed a normal rodent diet. p25 transgene expression was induced in adult mice at the age of 3 months.
Animal from both sexes were used, as indicated in the Methods.

Housing conditions: All animals are housed in groups of three to five in commercially available, individually ventilated cages (IVCs) made of Polysulfon under precisely defined standard laboratory conditions (room temperature 22 ± 1 °C; relative humidity 55 ± 10 %; photoperiod 12L:12D), supplied with standard diet (rat/mouse maintenance diet (V1534-300) or mouse breeding diet (V1124-300), ssniff Spezialitäten GmbH) and autoclaved water ad libitum.

Age: Adult (C57BL/6J) control and ovariectomized mice were 2-3 months old at time of perfusion. Cx3cr1GFP/- mice were sampled at postnatal days 7, 15, and 22. 5xFAD mice were collected at 3 and 6 months. After induction of p25 expression at age of 3 months, CK-p25 mice were collected after 1, 2 and 6 weeks. C57BL/6J mice were 2-3 months old upon administration of the anesthetic cocktail ketamine-xylazine-acepromazine (KXA).

Wild animals

No wild animals were used in this study.

Field-collected samples

No field-collected samples were used in this study.

Ethics oversight

IST Austria: All animal procedures are approved by the Bundesministerium für Wissenschaft, Forschung und Wirtschaft (bmwfw) Tierversuchsgesetz 2012, BGBI. I Nr. 114/2012, idF BGBI. I Nr. 31/2018 under the numbers 66.018/0005-WF/V/3b/2016, 66.018/0010-WF/V/3b/2017, 66.018/0025-WF/V/3b/2017, 66.018/0001_V/3b/2019, 2020-0.272.234.
MIT: All animal work was approved by the Committee for Animal Care of the Division of Comparative Medicine at the Massachusetts Institute of Technology.

Note that full information on the approval of the study protocol must also be provided in the manuscript.
